# Supplementary material for: The ORC ubiquitin ligase OBI1 promotes DNA replication origin firing
Source: Nat Commun. 2019 Jun 3;10:2426. doi: 10.1038/s41467-019-10321-x (PMC6547688; doi:10.1038/s41467-019-10321-x)
Supplement: Supplementary file 1 — Supplementary Information [file 41467_2019_10321_MOESM1_ESM.pdf]

ORC ubiquitin ligase OBI1 promotes DNA replication origin firing

Coulombe et al.

Supplementary Information

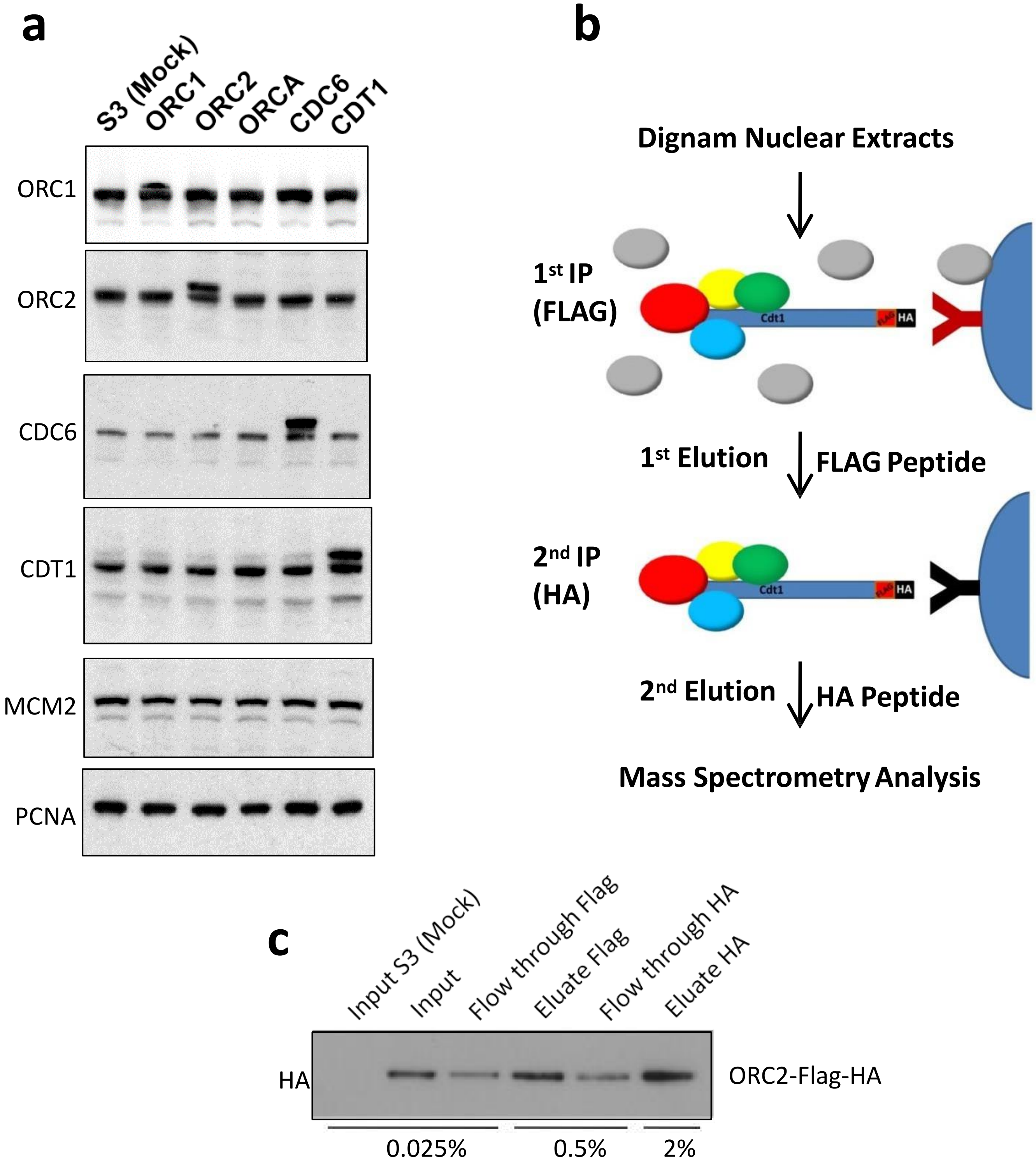

Supplementary Figure 1: Characterization of the TAP-TAG procedure

a) Expression level of pre-RC baits. Dignam extracts from parental HeLa S3 cells (Mock) and from cells transduced to express the indicated pre-RC factors were analysed by western blotting. **b)** Schematic representation of the TAP-TAG purification procedure. **c)** TAP-TAG purification efficiency. A Dignam extract from ORC2-Flag-HA expressing HeLa S3 cells was processed for TAP-TAG purification. Aliquots (in percentage) of fractions collected during the procedure were analysed by western blotting.

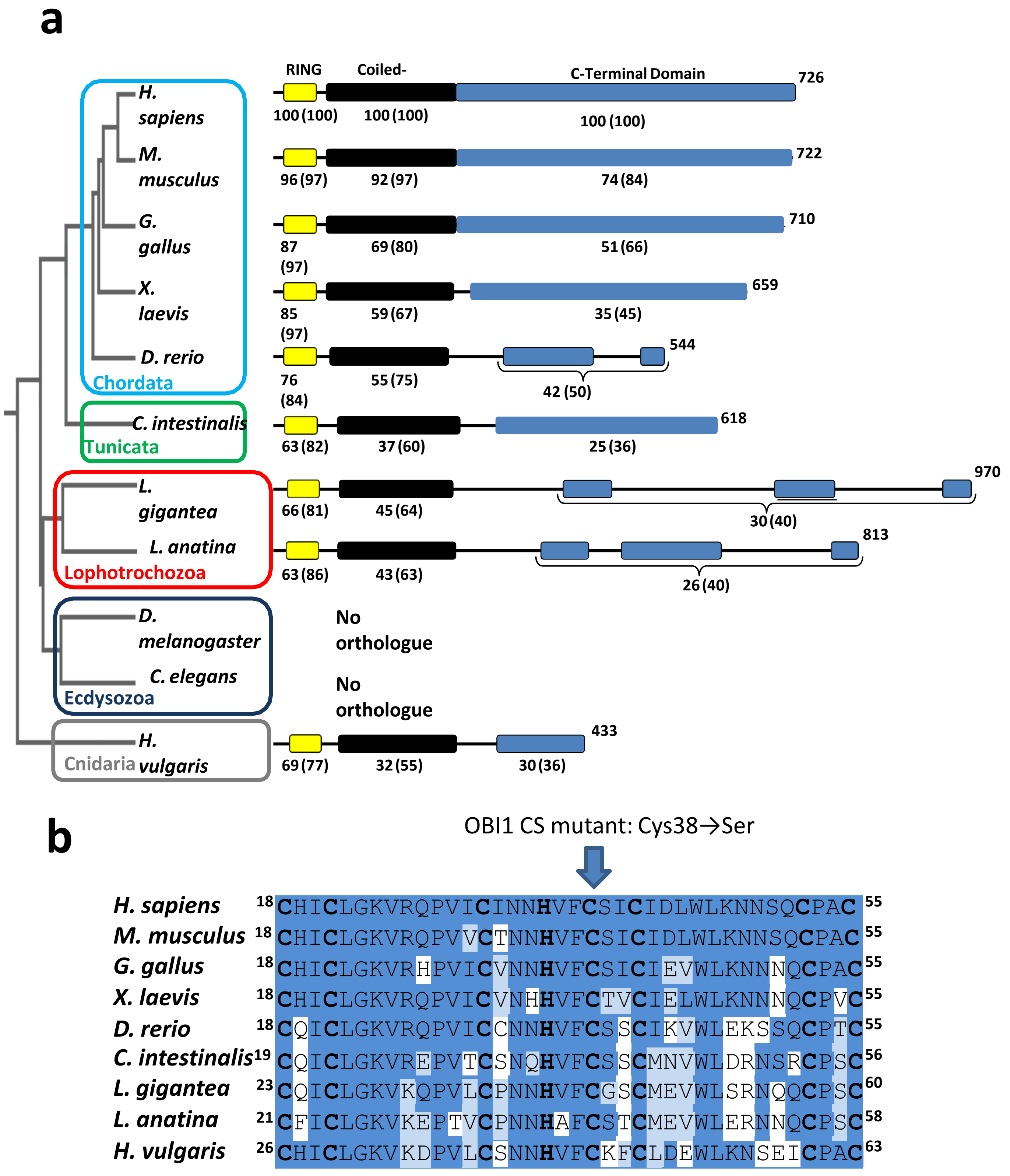

Supplementary Figure 2: OBI1 conservation

a, Phylogenetic tree showing the evolutionary relationship between species harbouring OBI1 homologues (left panel). Schematic representation of OBI1 homologues showing the conserved domains. The percentage of identity and similarity (in brackets) of OBI1 homologues compared with the human isoform is indicated. Phylogenetic tree and sequence alignments were generated using the SuperFamily (supfam.cs.bris.ac.uk) and BLAST (blast.ncbi.nlm.nih.gov) servers, respectively. **b,** Alignment of the RING domain of OBI1 homologues. Residues conserved relative to the human isoform are highlighted. Residues involved in coordinating the two zinc ions are in bold. The OBI1 RING domain point mutant Cys38Ser is highlighted.

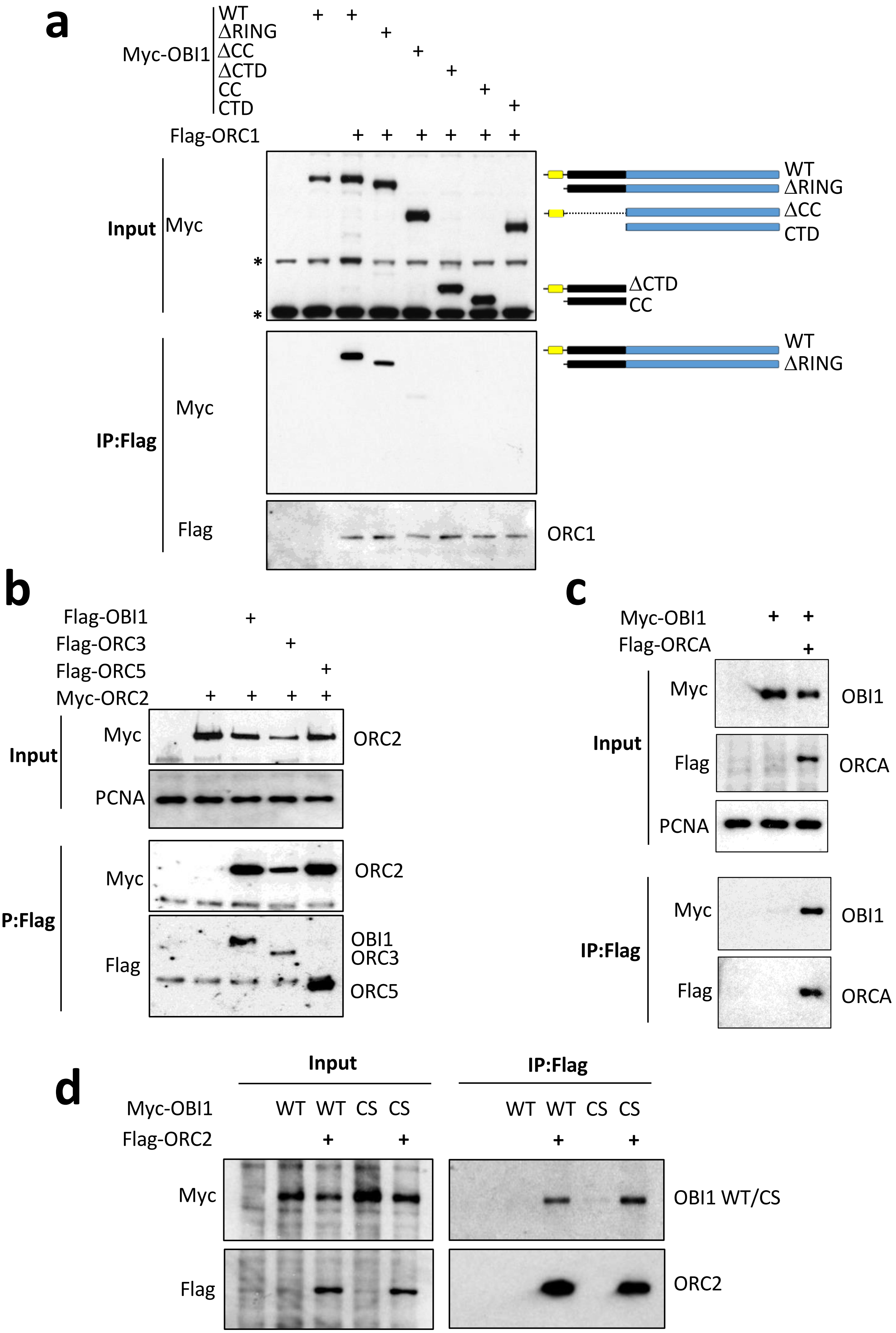

Supplementary Figure 3: Characterization of OBI1-ORC interaction

a-d, Association of OBI1 with the ORC complex. U2OS cells were transfected with tagged versions of ORC subunits and OBI1, as indicated. Input lysates and Flag-immunoprecipitates were analysed by western blotting.

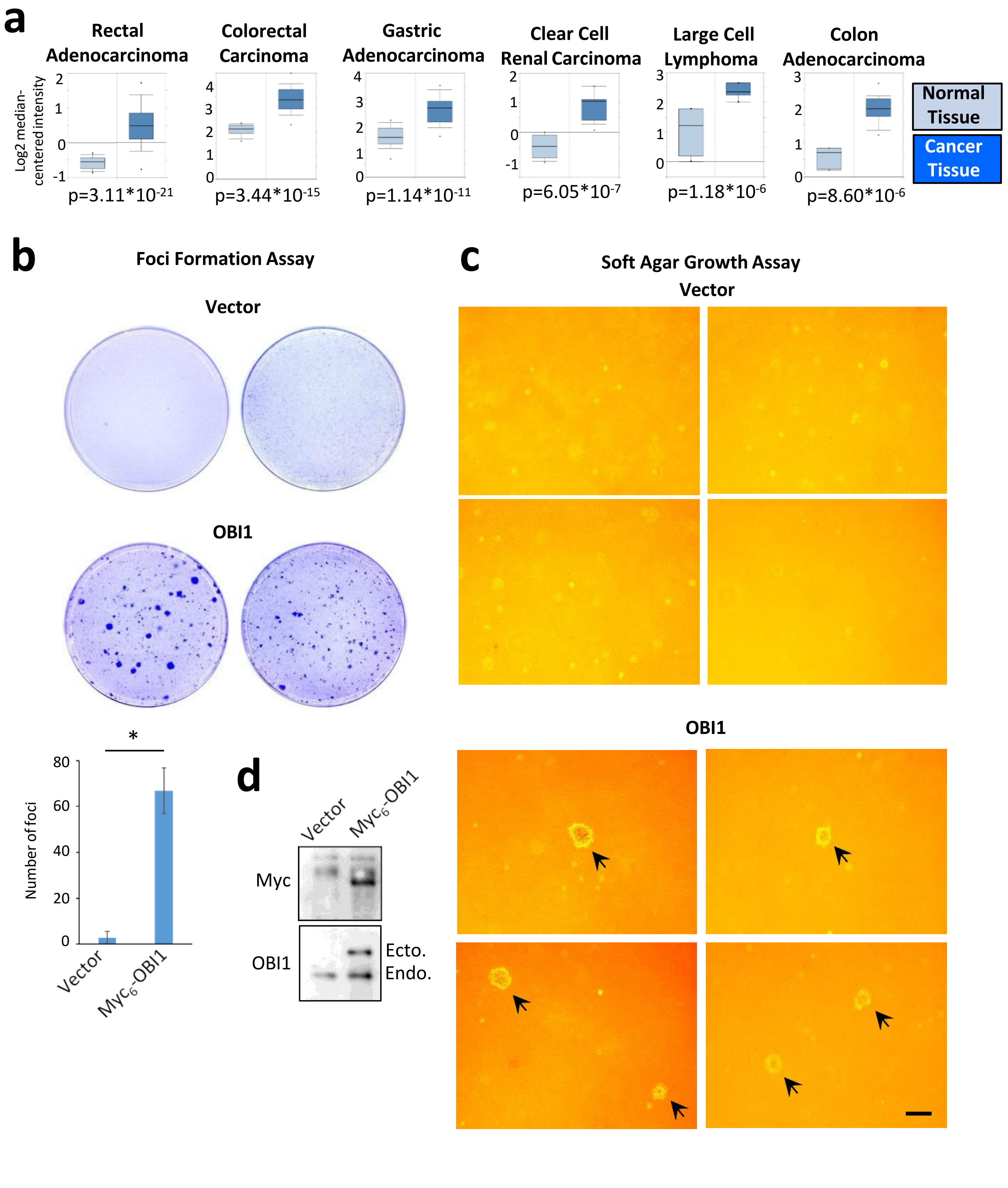

Supplementary Figure 4: OBI1 involvement in oncogenic transformation

a, OBI1 expression in human primary tumour samples and matched normal tissues was examined using the ONCOMINE server (oncomine.org). Cases in which expression was significantly different between tumour and normal tissue are shown. P-values are indicated. **b-d,** NIH 3T3 cells were stably transfected with empty vector or OBI1 expression plasmid and a puromycin-resistance plasmid. The oncogenic properties of cell populations were evaluated by foci formation assay (**b**, quantified in lower left panel) and soft agar growth assay (**c**). Arrows show OBI1-expressing colonies growing in soft agar. Scale bar, 300 mm. Endogenous (Endo) and ectopic (Ecto) OBI1 expression was evaluated in puromycin-selected cell cultures by western blotting (**d**).

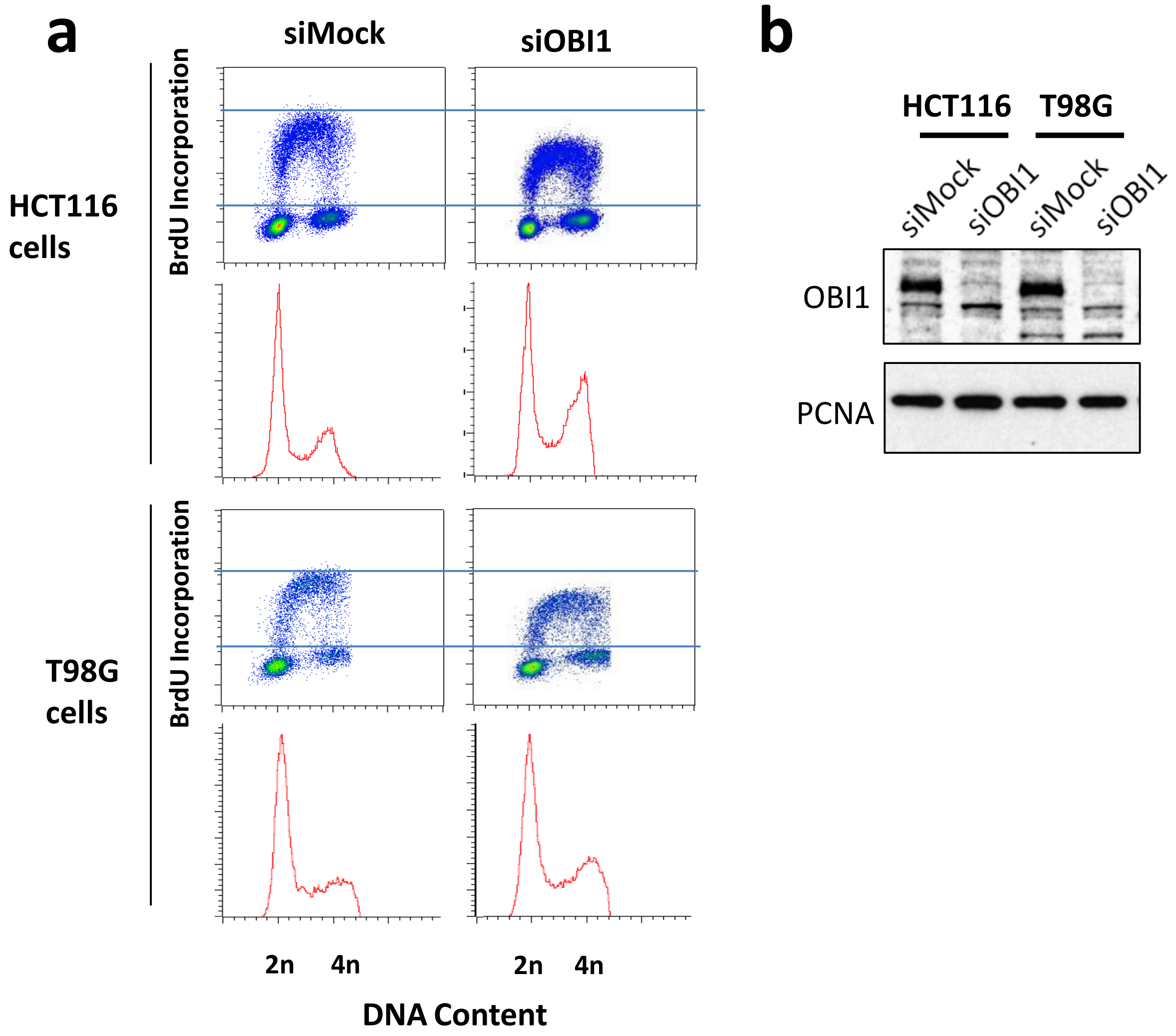

Supplementary Figure 5: OBI1 depletion in HCT116 and T98G cells results in DNA synthesis defects
a, Human colon cancer HCT116 and non-transformed hTERT-immortalized RPE1 cells were transfected with siRNAs targeting OBI1 (siOBI1) or mock (siMock), as indicated. Three days after transfection, cells were pulsed with BrdU for 15min and processed for flow cytometry analysis. Lines delimiting BrdU-positive siMock-treated cells are shown. **b,** Expression of endogenous OBI1 and PCNA was analysed in aliquots of cells described in **a** by western blotting.

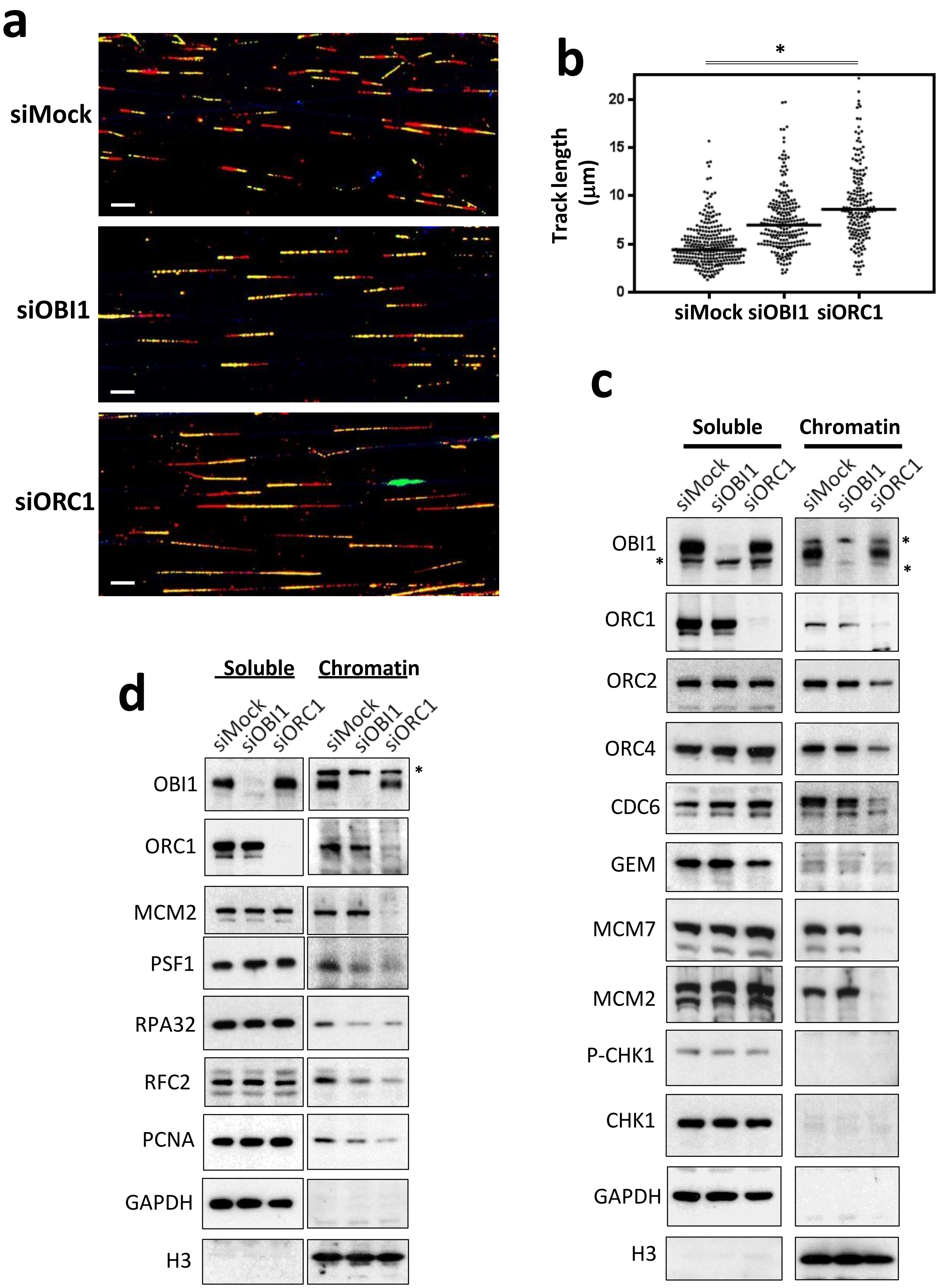

Supplementary Figure 6: OBI1 involvement in origin activation

a, DNA fibre stretching analysis. U2OS cells were transfected with Mock, *OBI1*- or *ORC1*-specific siRNAs. Three days later, cells were incubated with IdU (15min; red) followed by CldU (15min; green) and processed for DNA spreading. Representative images are shown. Scale bars, 5 mm. **b,** Fork speed analysis in Mock, *OBI1* and *ORC1* siRNA-transfected cells. The length of CldU tracks after the IdU signal was measured. More than 200 measurements are shown from two independent experiments. Bar indicates the median length. **c-d,** OBI1 is involved in origin firing, but not in origin licensing. U2OS cells were transfected with Mock, *OBI1*- or *ORC1*-specific siRNAs. Three days later, chromatin and soluble fractions were isolated and analysed by western blotting with antibodies against the indicated proteins. Asterisks mark non-specific bands.

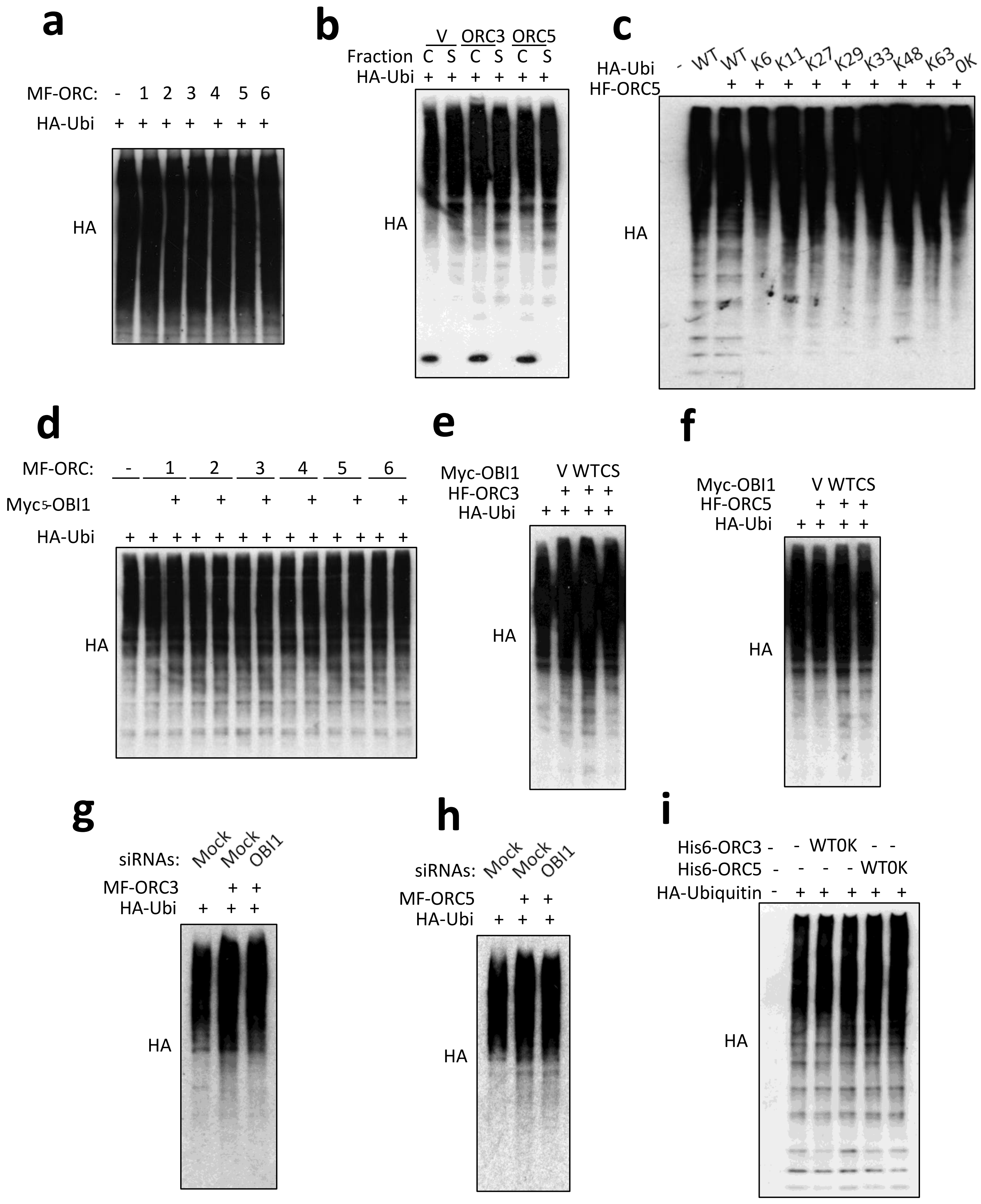

Supplementary Figure 7: Characterization of ORC ubiquitylation; complementary to Figure 3-5

a-c, Expression of HA-tagged ubiquitin in the experiments described in Figure 3a-c was evaluated by western blotting of input extracts. **d-h,** Expression of HA-tagged ubiquitin in the experiments described in Figure 4a-e was evaluated by western blotting of input extracts. **i,** Expression of HA-tagged ubiquitin in the experiment described in Figure 5a was evaluated by western blotting of input extracts.

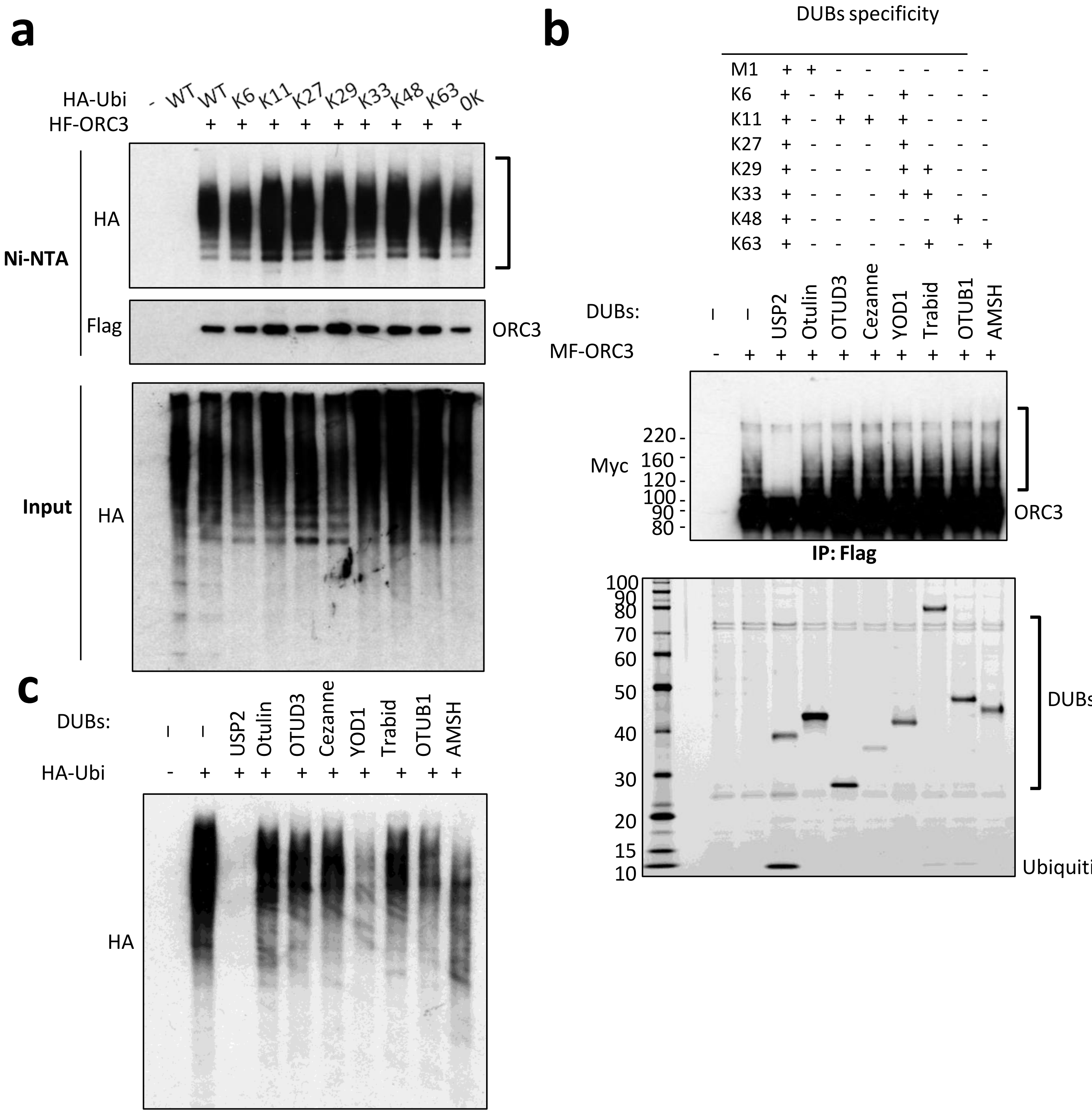

Supplementary Figure 8: Multi-mono-ubiquitylation of ORC3

a, U2OS cells were co-transfected with HF-ORC3 and the indicated HA-tagged ubiquitin single-lysine (K) mutants. In 0K ubiquitin, all lysine residues are replaced by arginine residues. Cells were lysed in denaturing conditions for His_6-tagged protein purification. Expression and ubiquitin conjugation of isolated proteins were monitored by western blotting, as indicated. **b,** UbiCRest analysis of ubiquitylated ORC3. U2OS cells were transfected with Myc-Flag (MF)-tagged ORC3 (A), without ectopic ubiquitin. Two days post-transfection, cells were lysed in NEM-containing buffer and Flag-tagged proteins were immunoprecipitated. After extensive washes, proteins bound to beads were incubated with the indicated deubiquitylating enzymes (DUBs), as specified by the manufacturer. At the end of the incubation, supernatants were recovered and analysed by silver staining. DUBs and released endogenous ubiquitin are indicated. Ubiquitylation was revealed by the presence of high molecular weight forms detected by western blotting against tagged-ORC3. **c,** UbiCRest assay on cellular ubiquitylated proteins. DUBs were incubated with extract expressing HA-ubiquitin as indicated. Ubiquitylation was revealed by anti-HA western blotting. Note the partial digestion of ubiquitylated proteins by several DUBs showing their activity.

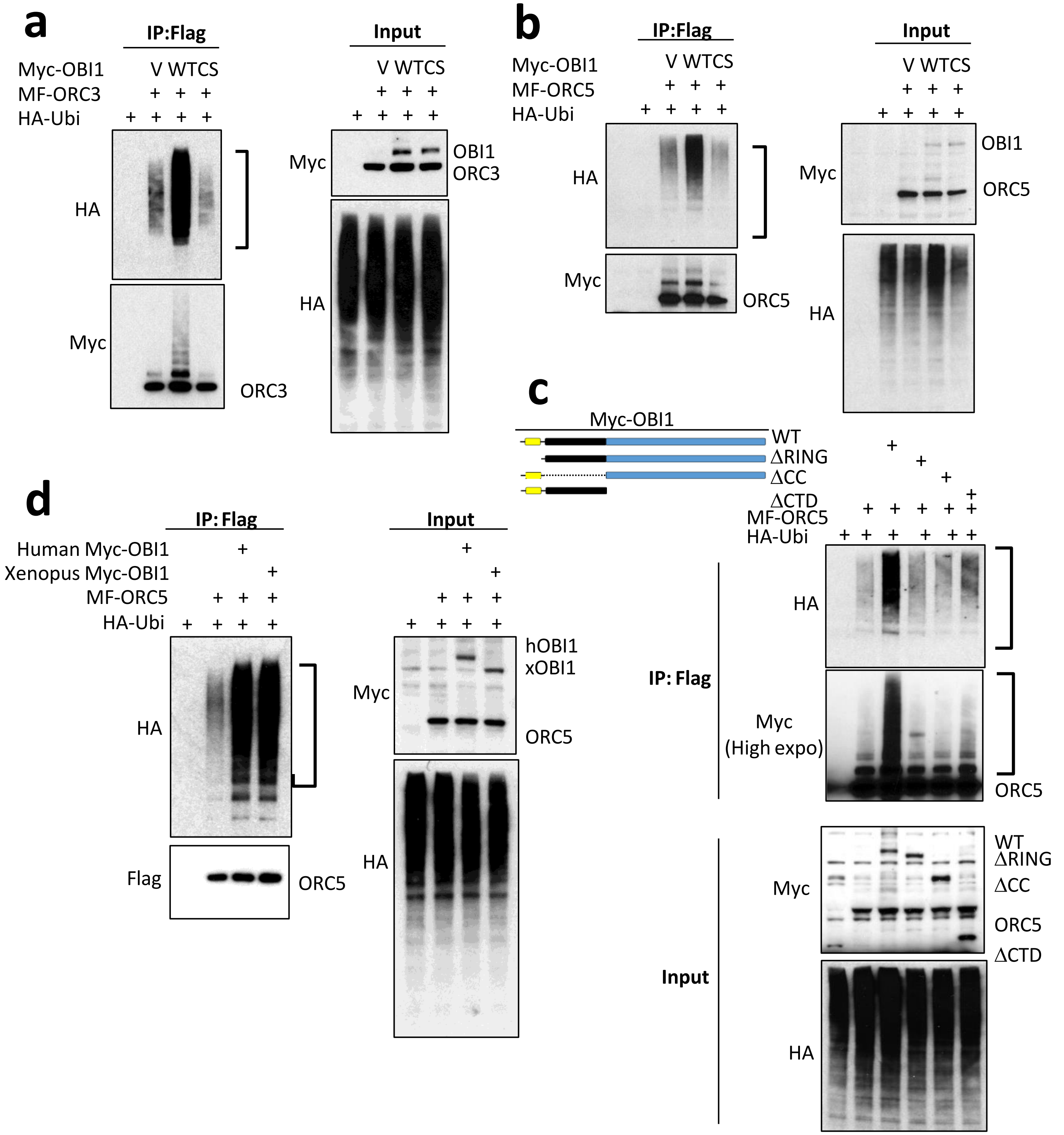

Supplementary Figure 9: Characterization of OBI1 ubiquitin ligase activity

a-b, U2OS cells were co-transfected with Myc-Flag (MF)-tagged ORC3 or ORC5, HA-ubiquitin and wild type (WT) OBI1 or the C38S (CS) mutant, as indicated. Two days post-transfection, cells were lysed in NEM-containing lysis buffer and tagged proteins were purified by immunoprecipitation. Expression and ubiquitin conjugation of the immunoprecipitates were monitored by western blotting, as indicated. Expression of tagged ORC and OBI1 was monitored in input extracts by western blotting. **c,** U2OS cells were co-transfected with Myc-Flag (MF)-ORC5 and the indicated OBI1 variants along with HA-ubiquitin. Two days post-transfection, cells were lysed in NEM-containing lysis buffer and ORC5 was purified by immunoprecipitation. Ubiquitin conjugation of immunoprecipitates and expression of ectopic proteins were monitored by western blotting, as indicated. **d,** U2OS cells were co-transfected with Myc-Flag (MF)-tagged ORC5 and human or *X. laevis* Myc-tagged OBI1 and HA-tagged ubiquitin. Two days post-transfection, cells were lysed in NEM-containing lysis buffer and ORC5 was purified by immunoprecipitation. Ubiquitin conjugation of immunoprecipitates and expression of ectopic proteins were monitored by western blotting, as indicated.

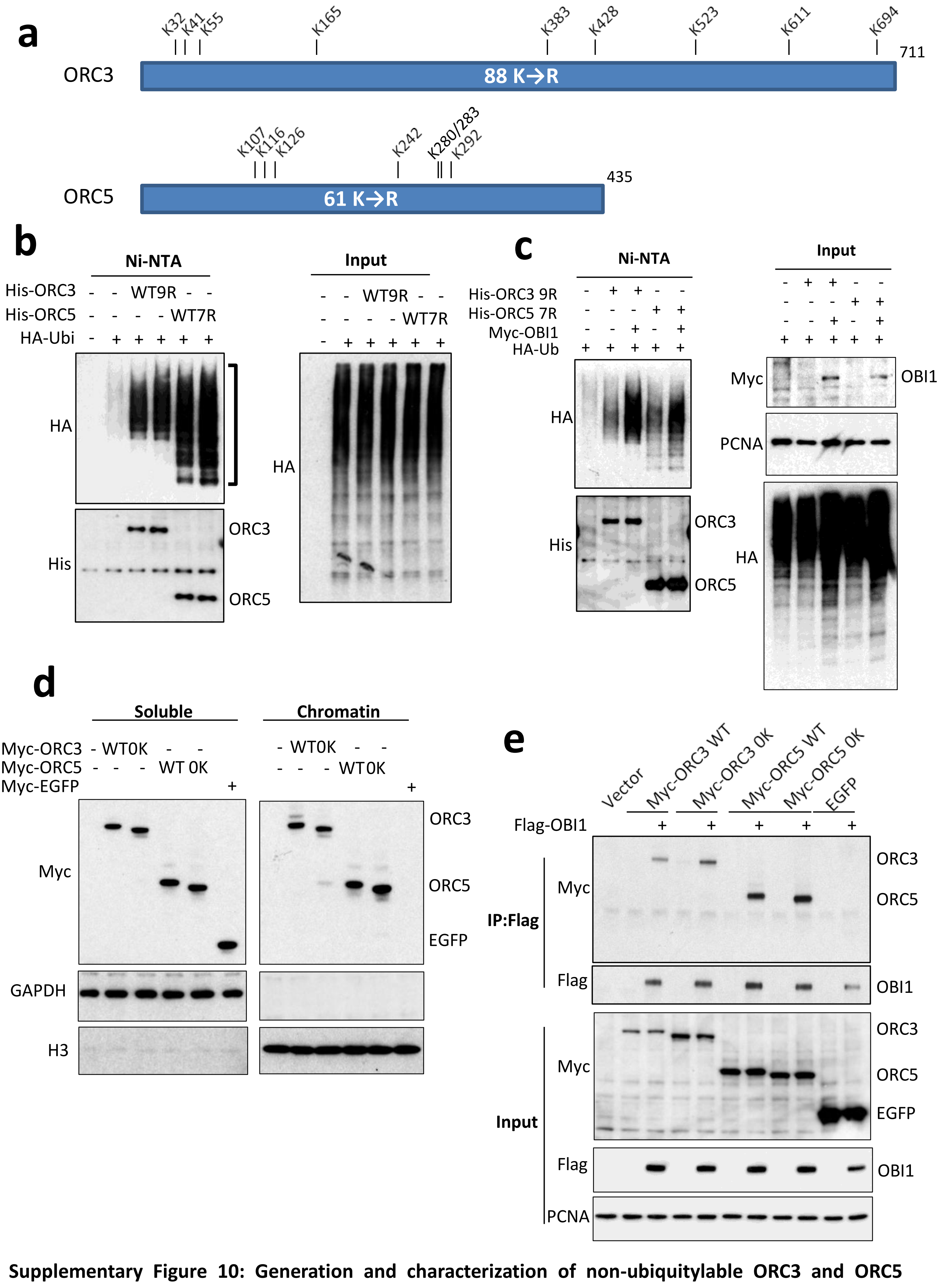

Supplementary Figure 10: Generation and characterization of non-ubiquitylable ORC3 and ORC5 variants

Supplementary Figure 10: Generation and characterization of non-ubiquitylable ORC3 and ORC5 variants

a, The lysine residues modified by ubiquitylation on ORC3 (n=9) and ORC5 (n=7) identified by large-scale mass spectrometry-based approaches (see main text for references) are highlighted. Also, the number of lysine to arginine (K→R) substitutions introduced in the 0K variant of ORC3 and ORC5 is indicated. **b,** U2OS cells were co-transfected with His_6-tagged wild type (WT) ORC3 and ORC5 or mutants for identified ubiquitylation sites (ORC3-9R and ORC5-7R) and with HA-ubiquitin, as indicated. Two days post-transfection, cells were lysed in denaturing conditions for His_6-tagged protein purification. Expression and ubiquitin conjugation of isolated proteins were monitored by western blotting, as indicated. **c,** U2OS cells were co-transfected with His_6-tagged-ORC3-9R and -ORC5-7R with Myc-OBI1 and HA-ubiquitin as indicated. Two days post-transfection, cells were lysed in denaturing conditions for His_6-tagged protein purification. Expression and ubiquitin conjugation of isolated proteins were monitored by western blotting, as indicated. **d,** U2OS cells were transfected with the indicated Myc-tagged ORC3 and ORC5 variants (WT or 0K) or EGFP as indicated. Two days after transfection, chromatin and soluble fractions were isolated and analysed by western blotting with the indicated antibodies. **e,** U2OS cells were co-transfected with the indicated Myc-tagged ORC3 and ORC5 variants (WT or 0K) or EGFP and with Flag-OBI1, as indicated. Two days after transfection, cell lysates were Flag-immunoprecipitated to purify the ORC subunit, and the association of co-expressed proteins was analysed by western blotting, as indicated. Expression of Myc-tagged proteins in input extracts was also monitored.

Supplementary Table 1: The human pre-RC interactome

ORC1		ORC2		LRWD1/ORCA		CDC6		CDT1	
**Protein**	**ΣPSM**	**Protein**	**ΣPSM**	**Protein**	**ΣPSM**	**Protein**	**ΣPSM**	**Protein**	**ΣPSM**
ORC1	312	ORC2	178	LRWD1/ORCA	358	CDC6	103	GEMININ	117
LRWD1/ORCA	86	ORC3	154	ORC1	108	DDX39B	9	CDT1	68
ORC2	85	LRWD1/ORCA	134	ORC3	101	LRWD1/ORCA	6	CDK1	28
ORC5	78	ORC4	81	ORC4	86	DDX5	6	CYCLIN A2	27
ORC4	73	ORC5	75	ORC5	76			CDK2	21
ORC3	72	FXR1	60	WIZ	66			SKP2	13
CYCLIN A2	64	ORC1	14	ORC2	65			SKP1	6
CULLIN-1	48	CYCLIN A2	6	ZNF644	55			CYCLIN A1	5
CDK2	46	XRCC6	5	EHMT1	43				
CDK1	37			HNRPM	30				
SKP2	30			RIF1	29				
XRCC5	19			EHMT2	29				
H4	16			FXR1	21				
XRCC6	14			CYCLIN A2	14				
TERF2	9			CDK2	13				
HNRPM	9			CDK1	10				
PSME3	9			CULLIN-1	9				
H2B	9			NUMA1	8				
OBI1/C13ORF7/RNF219	8			GEMININ	8				
TE2IP	8			SKP2	8				
RBM10	6			XRCC6	6				
H2A	6			PRMT5	6				
CKS1	5			TRIM29	6				
NFIL3	5			XRCC5	6				
SKP1	5			OBI1/C13ORF7/RNF219	5				
				UBIQUITIN	5				
				AKAP8	5				

List of the proteins identified by mass spectrometry as associated with the indicated baits. The total number of identified peptide sequences (peptide spectrum matches, ΣPSM) for each protein, including those redundantly identified, is indicated. Only proteins with ΣPSM ≥5 are shown.

Supplementary Table 2: Description of the human pre-RC interactome

Baits	Preys		
	Gene name	Description(s)	Function(s)
**ORC1**	ORC1	Origin recognition complex subunit 1	Pre-RC formation (origin licensing), AAA+ ATPase
	LRWD1 /ORCA	Leucine-rich WD40-containing protein 1 /ORC associated protein 1	ORC binding protein; Binds to heterochromatin epigenetic marks; heterochromatin formation
	ORC2	Origin recognition complex subunit 2	Pre-RC formation (origin licensing)
	ORC5	Origin recognition complex subunit 5	Pre-RC formation (origin licensing)
	ORC4	Origin recognition complex subunit 4	Pre-RC formation (origin licensing)
	ORC3	Origin recognition complex subunit 3	Pre-RC formation (origin licensing)
	CYCLIN A2	Cyclin A2	Binds and activates CDK; cell cycle regulation
	CULLIN-1	Cullin 1	SCF ubiquitin ligase complex; cell cycle regulation
	CDK2	Cyclin dependent kinase 2	Proline-directed kinase, cell cycle regulation
	CDK1	Cyclin dependent kinase 1	Proline-directed kinase, cell cycle regulation
	SKP2	S-phase kinase associated protein 2	SCF^{SKP2} ubiquitin ligase complex; cell cycle regulation
	XRCC5	X-ray repair cross complementing 5/Ku80	Associates with Ku70; DNA repair, Transcription
	H4	Histone H4	Nucleosome constituant
	XRCC6	X-ray repair cross complementing 6/Ku70	Associates with Ku80; DNA repair, Transcription
	TERF2	Telomeric repeat binding factor 2	Shelterin complex; telomere homeostasis
	HNRPM	Heterogeneous nuclear ribonucleoprotein M	RNA binding protein; mRNA metabolism
	PSME3	Proteasome activator subunit 3	Constituant of the proteasome 11S regulator
	H2B	Histone H2B	Nucleosome constituant
	OBI1/C13ORF7 /RNF219	ORC ubiquitin ligase 1/Chromosome 13 open reading frame 7/Ring finger protein 219	Previously uncharacterized; ORC complex ubiquitin ligase, promotes origin firing (present study)
	TE2IP	TERF2-interacting protein 2	Shelterin complex; telomere homeostasis
	RBM10	RNA binding motif protein 10	RNA binding protein; mRNA metabolism
	H2A	Histone H2A	Nucleosome constituant
	CKS1	Cyclin-dependant kinase regulatory subunit 1	Binds to CDK; cell cycle regulation
	NFIL3	Nuclear factor, interleukin 3 regulated	Transcription factor
	SKP1	S-phase kinase associated protein 1	SCF ubiquitin ligase complex; cell cycle regulation
**ORC2**	ORC2	Origin recognition complex subunit 2	Pre-RC formation (origin licensing)
	ORC3	Origin recognition complex subunit 3	Pre-RC formation (origin licensing)
	LRWD1 /ORCA	Leucine-rich WD40-containing protein 1 /ORC associated protein 1	ORC binding protein; Binds to heterochromatin epigenetic marks; heterochromatin formation

Supplementary Table 2: Description of the human pre-RC interactome

Baits	Preys		
	Gene name	Description(s)	Function(s)
**ORC2**	ORC4	Origin recognition complex subunit 4	Pre-RC formation (origin licensing)
	ORC5	Origin recognition complex subunit 5	Pre-RC formation (origin licensing)
	FXR1	FMR autosomal homolog 1	RNA binding protein, mRNA metabolism
	ORC1	Origin recognition complex subunit 1	Pre-RC formation (origin licensing), AAA+ ATPase
	CYCLIN A2	Cyclin A2	Binds and activates CDK; cell cycle regulation
	XRCC6	X-ray repair cross complementing 6/Ku70	Associates with Ku80; DNA repair, Transcription
**LRWD1 /ORCA**	LRWD1 /ORCA	Leucine-rich WD40-containing protein 1 /ORC associated protein 1	ORC binding protein; Binds to heterochromatin epigenetic marks; heterochromatin formation
	ORC1	Origin recognition complex subunit 1	Pre-RC formation (origin licensing), AAA+ ATPase
	ORC3	Origin recognition complex subunit 3	Pre-RC formation (origin licensing)
	ORC4	Origin recognition complex subunit 4	Pre-RC formation (origin licensing)
	ORC5	Origin recognition complex subunit 5	Pre-RC formation (origin licensing)
	WIZ	Widely Interspcaced Zinc Finger Motifs	Multiple Zinc finger protein, associates with EHMT1/2
	ORC2	Origin recognition complex subunit 2	Pre-RC formation (origin licensing)
	ZNF644	Zinc Finger Protein 644	Transcription factor, associates with EHMT1/2
	EHMT1	Euchromatic histone lysine methyltransferase 1	H3K9 methyltransferase, heterochromatin
	HNRPM	Heterogeneous nuclear ribonucleoprotein M	RNA binding protein; mRNA metabolism
	RIF1	Replication Timing Regulatory Factor 1	Nuclear matrix component, replication timing regulator
	EHMT2	Euchromatic histone lysine methyltransferase 2	H3K9 methyltransferase, heterochromatin
	FXR1	FMR autosomal homolog 1	RNA binding protein, mRNA metabolism
	CYCLIN A2	Cyclin A2	Binds and activates CDK; cell cycle regulation
	CDK2	Cyclin dependant kinase 2	Proline-directed kinase, cell cycle regulation
	CDK1	Cyclin dependant kinase 1	Proline-directed kinase, cell cycle regulation
	CULLIN-1	Cullin 1	SCF ubiquitin ligase complex; cell cycle regulation
	NUMA1	Nuclear Mitotic Apparatus Protein 1	Nuclear matrix component
	GEMININ	Geminin	CDT1 inhibitor, APC substrate
	SKP2	S-phase kinase associated protein 2	SCF^{SKP2} ubiquitin ligase complex; cell cycle regulation
	XRCC6	X-ray repair cross complementing 6/Ku70	Associates with Ku80; DNA repair, Transcription
	PRMT5	Protein Arginine Methyltransferase 5	Transcription regulation, ribonucleoprotein assembly
	TRIM29	Tripartite Motif Containing 29	Zinc Finger protein, transcription regulation
	XRCC5	X-ray repair cross complementing 5/Ku80	Associates with Ku70; DNA repair, Transcription

Supplementary Table 2: Description of the human pre-RC interactome

Baits	Preys		
	Gene name	Description(s)	Function(s)
**LRWD1 /ORCA**	OBI1/C13ORF7 /RNF219	ORC-ubiquitin-ligase-1/Chromosome 13 Open Reading Frame 7/RING Finger Protein 219	Previously uncharacterized; ORC complex ubiquitin ligase, promotes origin firing (present study)
	UBIQUITIN	Ubiquitin	Protein degradation, signaling
	AKAP8	A-Kinase Anchoring Protein 8	Bind PKA, associates with nuclear matrix
**CDC6**	CDC6	Cell Division Cycle 6	Pre-RC formation (origin licensing), AAA+ ATPase
	DDX39B	DExD-box Helicase 39B	ATP-dependant RNA helicase, mRNA splicing and export
	LRWD1 /ORCA	Leucine-rich WD40-containing protein 1 /ORC associated protein 1	ORC binding protein; Binds to heterochromatin epigenetic marks; heterochromatin formation
	DDX5	DExD-box Helicase 39B	ATP-dependant RNA helicase
**CDT1**	GEMININ	Geminin	CDT1 inhibitor, APC substrate
	CDT1	CDC10 Dependent Transcript 1	Pre-RC formation (origin licensing)
	CDK1	Cyclin dependant kinase 1	Proline-directed kinase, cell cycle regulation
	CYCLIN A2	Cyclin A2	Binds and activates CDK; cell cycle regulation
	CDK2	Cyclin dependant kinase 2	Proline-directed kinase, cell cycle regulation
	SKP2	S-phase kinase associated protein 2	SCF^{SKP2} ubiquitin ligase complex; cell cycle regulation
	SKP1	S-phase kinase associated protein 2	SCF ubiquitin ligase complex; cell cycle regulation
	CYCLIN A1	Cyclin A1	Binds and activates CDK; cell cycle regulation

Supplementary Table 3: Sequence of siRNAs used in this study

siRNAs	Sequences
siMock	UAAGGCUAUGAAGAGAUAC
siOBI1	GUAGAAGUAAUGUUAGAUG<br>CCAAAGGUUCUCUAACUAA<br>GGAUUUGGAUGGGUUAUCA<br>GAACGAAGUGAUAAGUAUA
siUTR	GCACUUGGGUUUAGAGGAA<br>GUCAGGAUGGUGGAUUAAC
siCDC7	CAGGAAAGGUGUUCACAAA<br>CUACACAAAUGCACAAAUU<br>GUACGGGAAUAUAUGCUUA<br>GCAUUCAUCAGUUUGGUAU
siORC1	UAAGAAACGUGCUCGAGUA<br>GAGAUCACCUCACCUUCUA<br>GCAGAGAGCCCUUCUUGGA<br>CCUCAGAUCCGUAGUCGAA

Supplementary Discussion: Information on the known interactors found associated with pre-RC components (related to Fig. 1a,b and Supplementary Table 1 and 2)

The ORC complex. All ORC subunits (with the exception of ORC6, see below) were associated with the baits ORC1, ORC2 and ORCA (Figure 1A-C and Table 1). Tagged ORCA strongly associated with the ORC subunits, while the endogenous protein was found in ORC1 and ORC2 complexes, confirming previous reports that ORCA is a *bona fide* additional ORC subunit [1-3]. Figures 1a-c and Supplementary Table 1 and 2 show ORC2 presence in ORC1 and ORCA baits, and ORC1 presence in ORC2 and ORCA baits specifically. The amount of ORC1 was lower in purified ORC2 and ORCA complexes. We think that the main reason is the known fate of ORC1 and ORC2 during the cell cycle in mammals [4]. In contrast to yeast in which all six ORC subunits are stably bound to chromatin throughout the cell cycle [5-9], the affinity of mammalian ORC1 for chromatin is selectively reduced during S-phase. Thus, ORC1 binds to chromatin during G1, and is then largely released from chromatin during S- [10] and M-phase [11], whereas ORC2 remains constantly bound to chromatin during S-phase. Therefore, in exponentially growing cells (our experimental condition in Fig. 1c), ORC2 will be in excess relative to ORC1.
ORC6 was not associated with ORC1, ORC2 or ORCA using our purification conditions. This finding could be explained by the fact that the ORC1-5/ORC6 interaction is salt-sensitive [12] and that our nuclear extraction was performed in high-salt buffer (see Methods section). This could also explain why we observed few interactions with the CDC6 bait.

Geminin. Geminin is an important negative regulator of origin licensing through direct binding to and inhibition of CDT1 [13]. Accordingly, endogenous geminin strongly and specifically interacted with the CDT1 bait (Fig. 1c and Supplementary Table 1 and 2). Low amount of geminin was also associated with the ORCA bait (Fig. 1c and Supplementary Table 1 and 2), as previously reported [14].

Cyclin-dependent kinases (CDKs). CDKs are important regulators of origin licensing and activation (for review [17]). CDKs (CDK1 and CDK2) as well as regulatory (cyclin A1 and A2) and accessory (CKS1) subunits were associated with the ORC complex and CDT1 baits (Fig. 1a-c and Supplementary Table 1 and 2). Both ORC1 and CDT1, which directly interact with cyclin A through their cyclin binding (Cy) motif [15,16], associated with significant amount of cyclin A. ORC2 and ORCA complexes included less ORC1 and also contained significantly lower amounts of cyclin A (Fig. 1c).

SCF^{Skp2} ubiquitin ligase. ORC1 and CDT1 are negatively regulated via degradation [17] by the cullin-based SCF^{Skp2} ubiquitin ligase [18-20]. In agreement, components of this E3 ligase were associated with the ORC1, ORCA and CDT1 baits (Fig. 1a,b and Supplementary Table 1 and 2).

Shelterin complex. The shelterin complex plays an important role in ensuring telomere integrity and genome stability [21]. TERF2 and TE2IP bind to telomere repetitive DNA and recruit the shelterin complex to telomeres. TERF2 also recruits the ORC complex to telomeres, thus facilitating telomere replication and stability. As previously reported [22,23], ORC1 specifically associated with the shelterin complex subunits TERF2 and TE2IP (Fig. 1a,b and Supplementary Table 1 and 2).

G9a/b H3K9 methyltransferase complexes. The ORC complex associates with heterochromatin marks, including H3K9me3 and H3K27me3 [2,3]. ORCA plays a role in recruiting the ORC complex to heterochromatin [24]. It was shown recently that ORCA interacts with the H3K9 methyltransferase (HMT) G9a/b [24], and is important for heterochromatin formation and histone methyltransferase stability. We also observed that the ORCA bait strongly interacted with several subunits of this HMT complex (G9a, G9b, WIZ and ZNF644) (Fig. 1a,b and Supplementary Table 1 and 2). WIZ and ZNF644 facilitate G9a/b interaction with chromatin [25].

Ku proteins. The heterodimeric Ku proteins Ku70 and Ku86 are DNA binding proteins involved in many chromatin transactions, notably in DNA repair pathways [26]. It is reported that Ku70/86 could play a role in DNA replication, by interacting with the ORC complex and facilitating origin establishment [27,28]. Here, we observed a specific association of Ku70 and Ku86 with the ORC1 and ORCA baits (Fig. 1a,b and Supplementary Table 1 and 2).

Histones. The association between histones and ORC1 observed in our pre-RC interactome study (Fig. 1a,b and Supplementary Table 1 and 2) could be related to the finding that ORC1, through its BAH domain, directly interacts with histone K4 dimethylated on lysine 20 (H4K20me2) and the nucleosome [29,30].

SUPPLEMENTARY REFERENCES

1 Shen, Z. *et al.* A WD-repeat protein stabilizes ORC binding to chromatin. *Mol Cell* **40**, 99-111, doi:10.1016/j.molcel.2010.09.021 (2010).

2 Bartke, T. *et al.* Nucleosome-interacting proteins regulated by DNA and histone methylation. *Cell* **143**, 470-484, doi:10.1016/j.cell.2010.10.012 (2010).

3 Vermeulen, M. *et al.* Quantitative interaction proteomics and genome-wide profiling of epigenetic histone marks and their readers. *Cell* **142**, 967-980, doi:10.1016/j.cell.2010.08.020 (2010).

4 Li, C. J. & DePamphilis, M. L. Mammalian Orc1 protein is selectively released from chromatin and ubiquitinated during the S-to-M transition in the cell division cycle. *Mol Cell Biol* **22**, 105-116 (2002).

5 Diffley, J. F., Cocker, J. H., Dowell, S. J. & Rowley, A. Two steps in the assembly of complexes at yeast replication origins in vivo. *Cell* **78**, 303-316 (1994).

6 Fujita, M. *et al.* Cell cycle dependent topological changes of chromosomal replication origins in Saccharomyces cerevisiae. *Genes Cells* **3**, 737-749 (1998).

7 Kong, D. & DePamphilis, M. L. Site-specific DNA binding of the Schizosaccharomyces pombe origin recognition complex is determined by the Orc4 subunit. *Mol Cell Biol* **21**, 8095-8103, doi:10.1128/MCB.21.23.8095-8103.2001 (2001).

8 Liang, C. & Stillman, B. Persistent initiation of DNA replication and chromatin-bound MCM proteins during the cell cycle in cdc6 mutants. *Genes & development* **11**, 3375-3386 (1997).

9 Lygerou, Z. & Nurse, P. The fission yeast origin recognition complex is constitutively associated with chromatin and is differentially modified through the cell cycle. *Journal of cell science* **112 (Pt 21)**, 3703-3712 (1999).

10 Kreitz, S., Ritzi, M., Baack, M. & Knippers, R. The human origin recognition complex protein 1 dissociates from chromatin during S phase in HeLa cells. *The Journal of biological chemistry* **276**, 6337-6342, doi:10.1074/jbc.M009473200 (2001).

11 Natale, D. A., Li, C. J., Sun, W. H. & DePamphilis, M. L. Selective instability of Orc1 protein accounts for the absence of functional origin recognition complexes during the M-G(1) transition in mammals. *The EMBO journal* **19**, 2728-2738, doi:10.1093/emboj/19.11.2728 (2000).

12 Ghosh, S., Vassilev, A. P., Zhang, J., Zhao, Y. & DePamphilis, M. L. Assembly of the human origin recognition complex occurs through independent nuclear localization of its components. *The Journal of biological chemistry* **286**, 23831-23841, doi:10.1074/jbc.M110.215988 (2011).

13 McGarry, T. J. & Kirschner, M. W. Geminin, an inhibitor of DNA replication, is degraded during mitosis. *Cell* **93**, 1043-1053 (1998).

14 Shen, Z. *et al.* Dynamic association of ORCA with prereplicative complex components regulates DNA replication initiation. *Mol Cell Biol* **32**, 3107-3120, doi:10.1128/MCB.00362-12 (2012).

15 Hossain, M. & Stillman, B. Meier-Gorlin syndrome mutations disrupt an Orc1 CDK inhibitory domain and cause centrosome reduplication. *Genes & development* **26**, 1797-1810, doi:10.1101/gad.197178.112 (2012).
(2012).

15 Hossain, M. & Stillman, B. Meier-Gorlin syndrome mutations disrupt an Orc1 CDK inhibitory domain and cause centrosome reduplication. *Genes & development* **26**, 1797-1810, doi:10.1101/gad.197178.112 (2012).
16 Sugimoto, N. *et al.* Cdt1 phosphorylation by cyclin A-dependent kinases negatively regulates its function without affecting geminin binding. *The Journal of biological chemistry* **279**, 19691-19697, doi:10.1074/jbc.M313175200 (2004).
17 Masai, H., Matsumoto, S., You, Z., Yoshizawa-Sugata, N. & Oda, M. Eukaryotic chromosome DNA replication: where, when, and how? *Annu Rev Biochem* **79**, 89-130, doi:10.1146/annurev.biochem.052308.103205 (2010).
18 Mendez, J. *et al.* Human origin recognition complex large subunit is degraded by ubiquitin-mediated proteolysis after initiation of DNA replication. *Mol Cell* **9**, 481-491 (2002).
19 Li, X., Zhao, Q., Liao, R., Sun, P. & Wu, X. The SCF(Skp2) ubiquitin ligase complex interacts with the human replication licensing factor Cdt1 and regulates Cdt1 degradation. *The Journal of biological chemistry* **278**, 30854-30858, doi:10.1074/jbc.C300251200 (2003).
20 Nishitani, H. *et al.* Two E3 ubiquitin ligases, SCF-Skp2 and DDB1-Cul4, target human Cdt1 for proteolysis. *The EMBO journal* **25**, 1126-1136, doi:10.1038/sj.emboj.7601002 (2006).
21 Hockemeyer, D. & Collins, K. Control of telomerase action at human telomeres. *Nat Struct Mol Biol* **22**, 848-852, doi:10.1038/nsmb.3083 (2015).
22 Deng, Z., Norseen, J., Wiedmer, A., Riethman, H. & Lieberman, P. M. TERRA RNA binding to TRF2 facilitates heterochromatin formation and ORC recruitment at telomeres. *Mol Cell* **35**, 403-413, doi:10.1016/j.molcel.2009.06.025 (2009).
23 Tatsumi, Y. *et al.* Involvement of human ORC and TRF2 in pre-replication complex assembly at telomeres. *Genes Cells* **13**, 1045-1059, doi:10.1111/j.1365-2443.2008.01224.x (2008).
24 Giri, S. & Prasanth, S. G. Association of ORCA/LRWD1 with repressive histone methyl transferases mediates heterochromatin organization. *Nucleus* **6**, 435-441, doi:10.1080/19491034.2015.1102814 (2015).
25 Bian, C., Chen, Q. & Yu, X. The zinc finger proteins ZNF644 and WIZ regulate the G9a/GLP complex for gene repression. *eLife* **4**, doi:10.7554/eLife.05606 (2015).
26 Fell, V. L. & Schild-Poulter, C. The Ku heterodimer: function in DNA repair and beyond. *Mutat Res Rev Mutat Res* **763**, 15-29, doi:10.1016/j.mrrev.2014.06.002 (2015).
27 Sibani, S., Price, G. B. & Zannis-Hadjopoulos, M. Ku80 binds to human replication origins prior to the assembly of the ORC complex. *Biochemistry* **44**, 7885-7896, doi:10.1021/bi047327n (2005).
28 Sibani, S., Price, G. B. & Zannis-Hadjopoulos, M. Decreased origin usage and initiation of DNA replication in haploinsufficient HCT116 Ku80+/- cells. *Journal of cell science* **118**, 3247-3261, doi:10.1242/jcs.02427 (2005).
29 Kuo, A. J. *et al.* The BAH domain of ORC1 links H4K20me2 to DNA replication licensing and Meier-Gorlin syndrome. *Nature* **484**, 115-119 (2012).
30 Zhang, W., Sankaran, S., Gozani, O. & Song, J. A Meier-Gorlin syndrome mutation impairs the ORC1-nucleosome association. *ACS Chem Biol* **10**, 1176-1180, doi:10.1021/cb5009684 (2015).

For Figure 2- g:

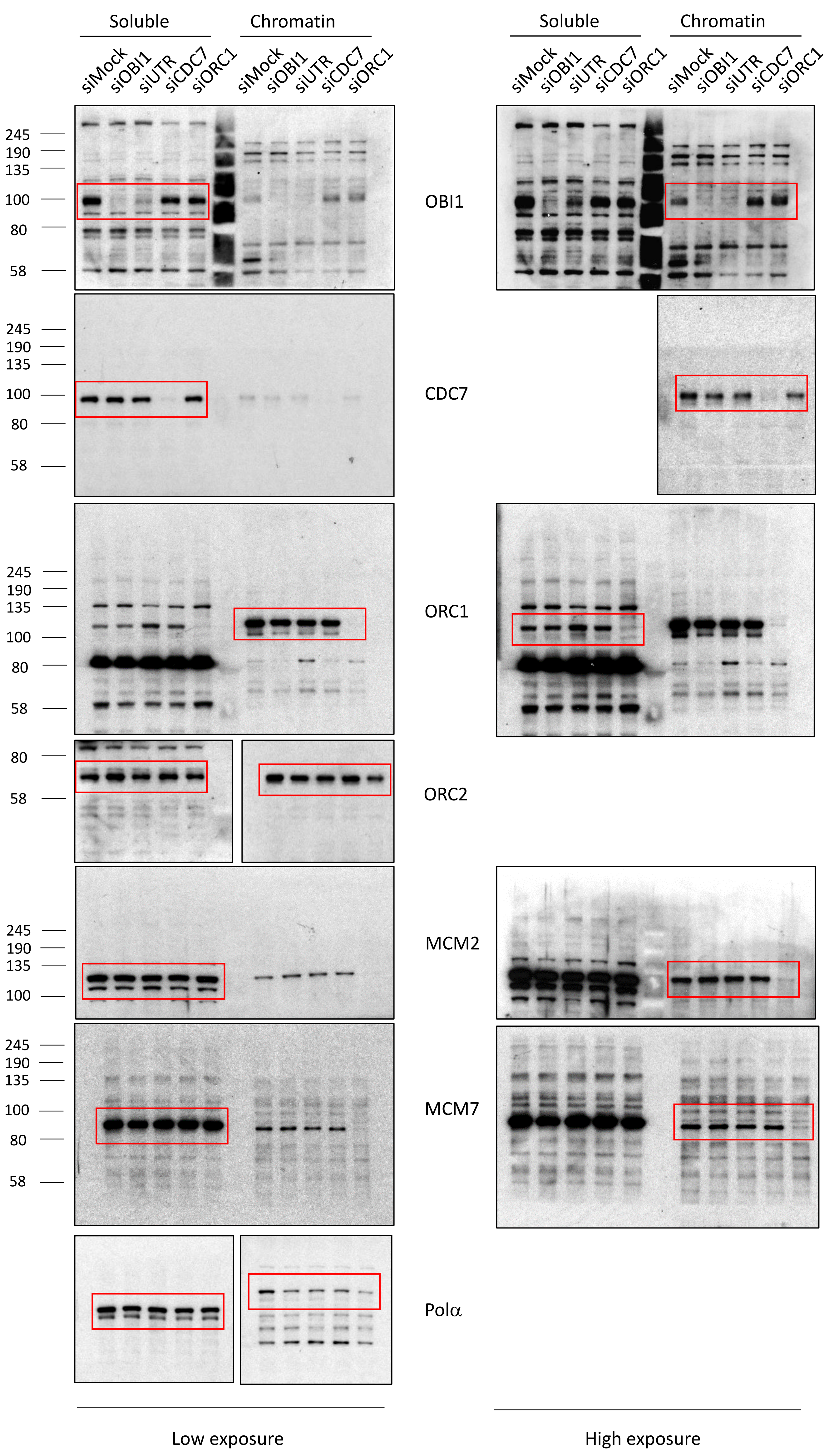

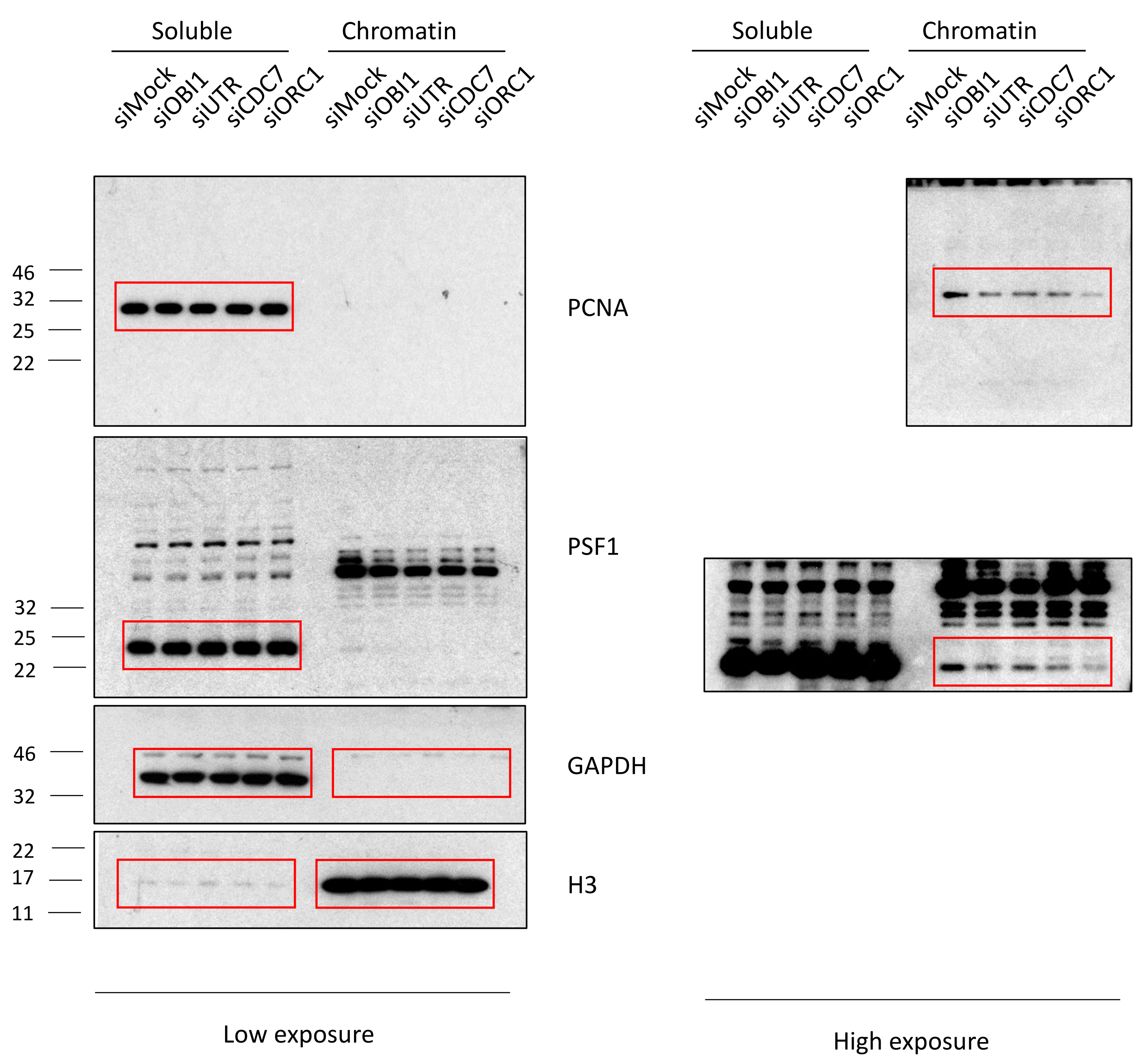

Supplementary Figure 11: Uncropped blots corresponding to Figure 2

SDS polyacrylamide gels for western blots found in Figure 2- g. Gels were loaded with the same samples from the same experiment.
Boxed areas correspond to the images presented in the main text.

For Figure 3- a:

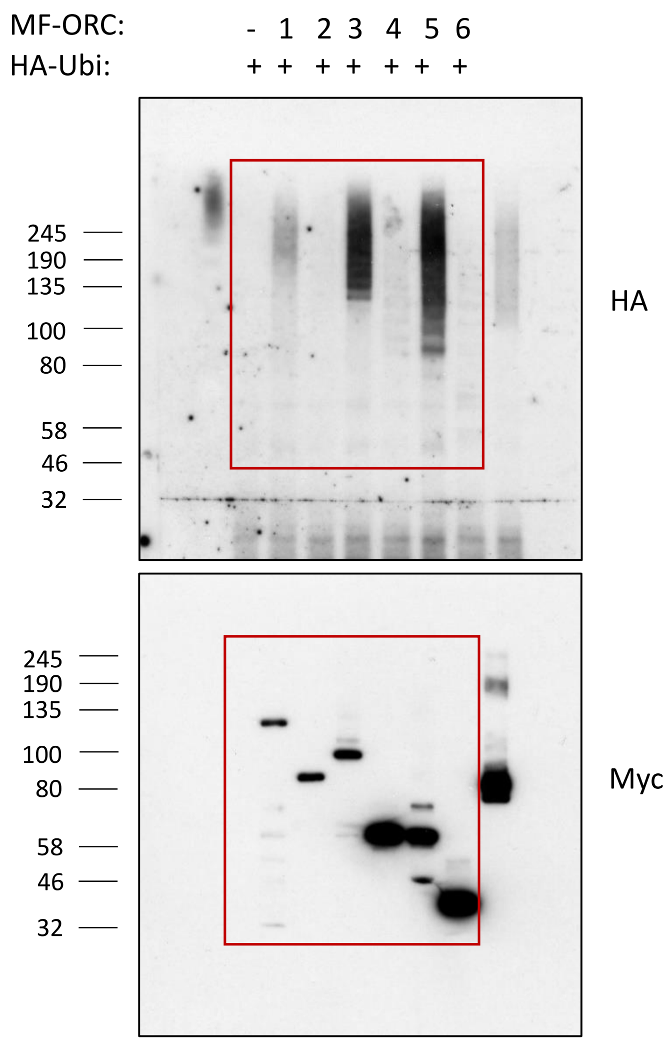

For Figure 3- b:

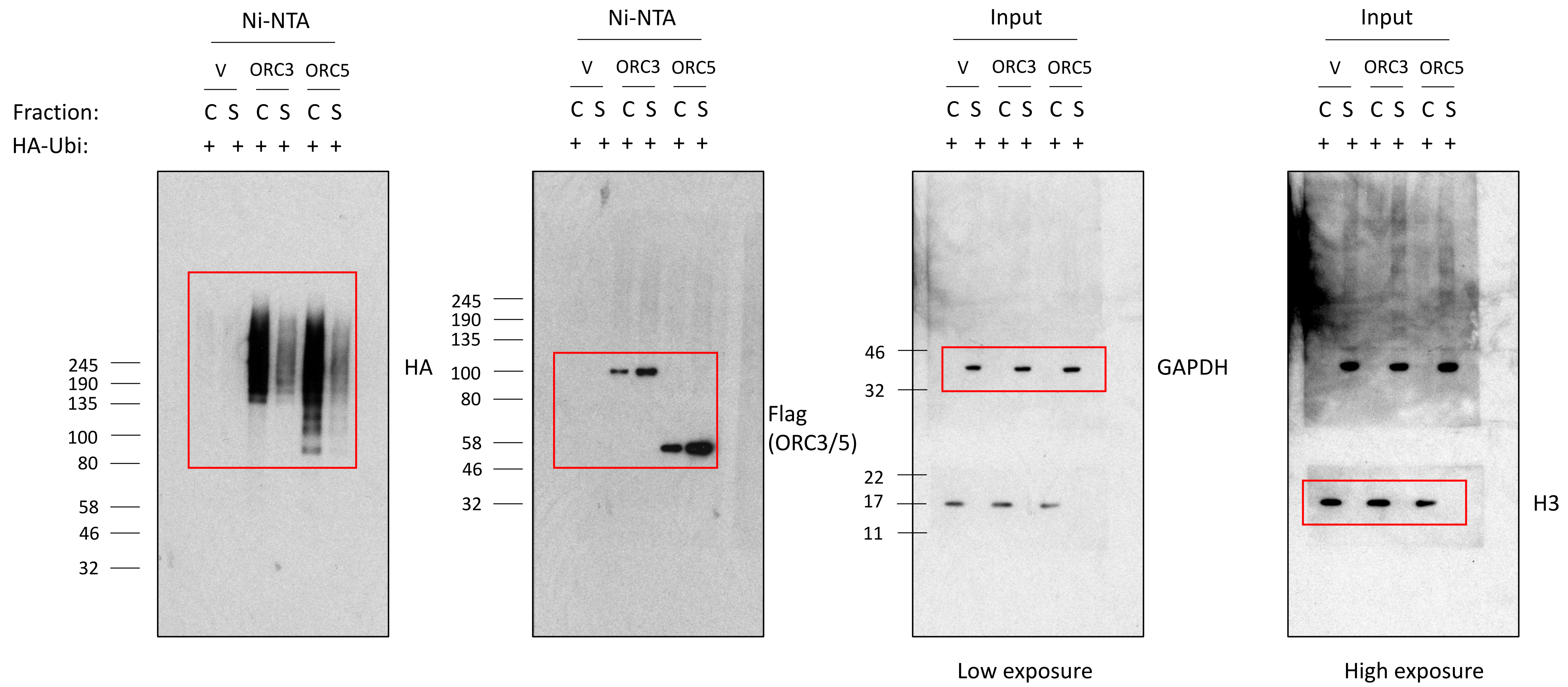

For Figure 3- c:

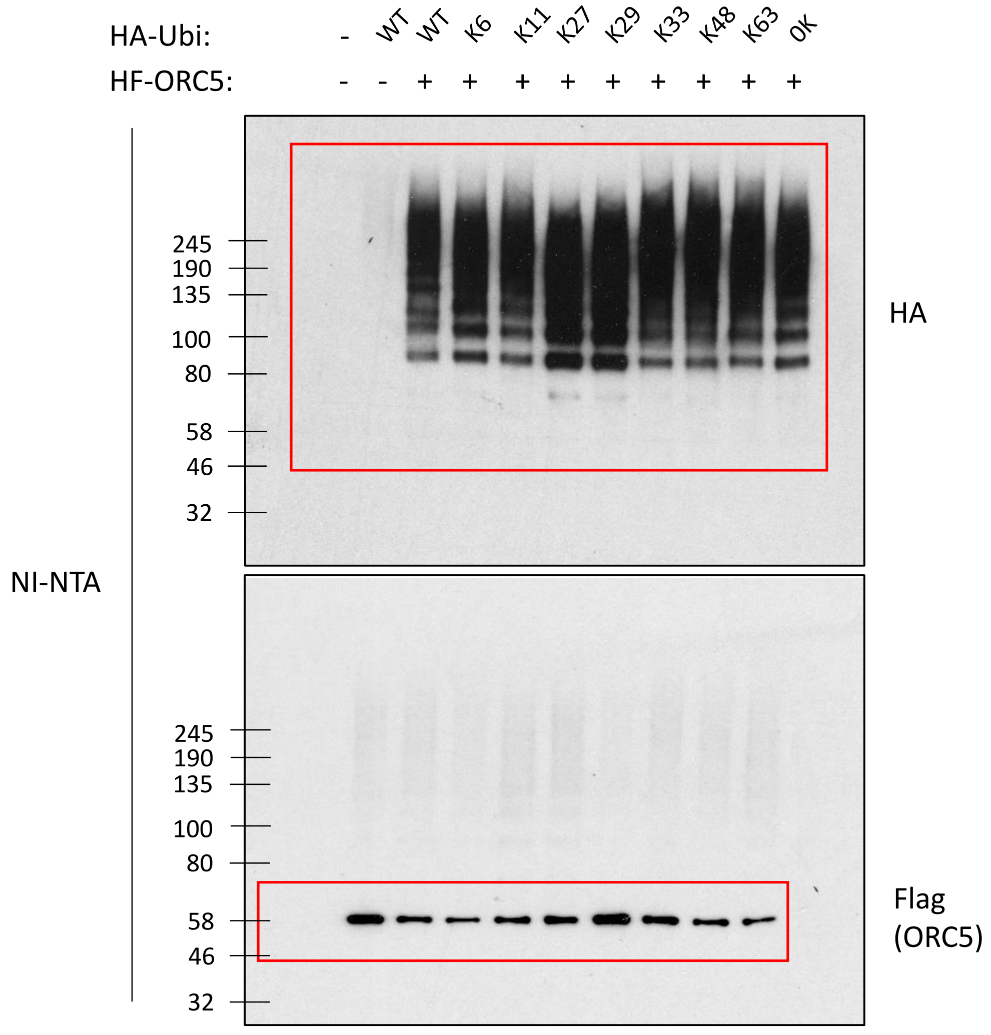

For Figure 3- d:

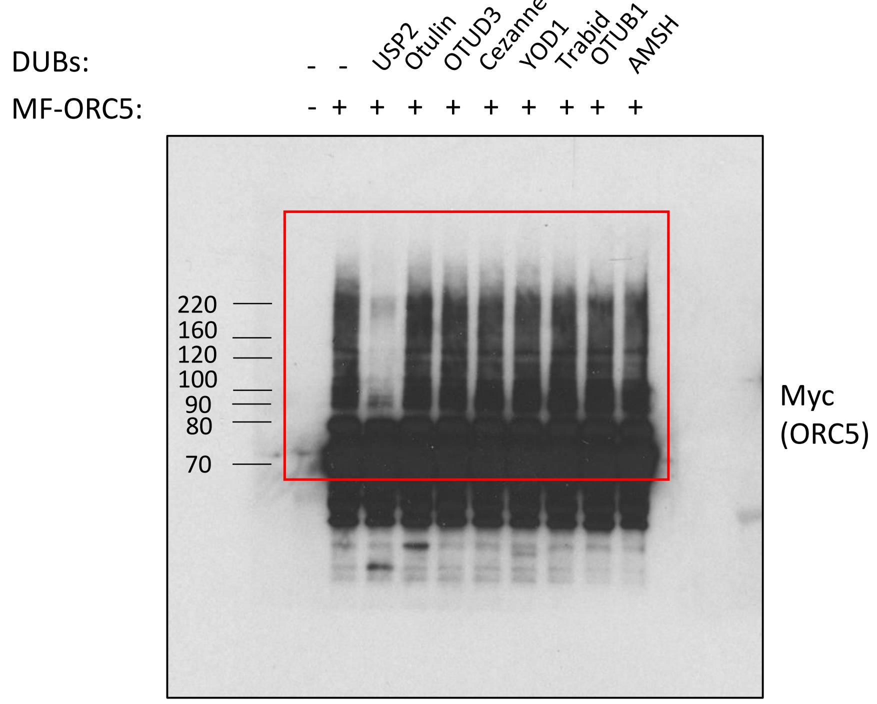

For Figure 3- e:

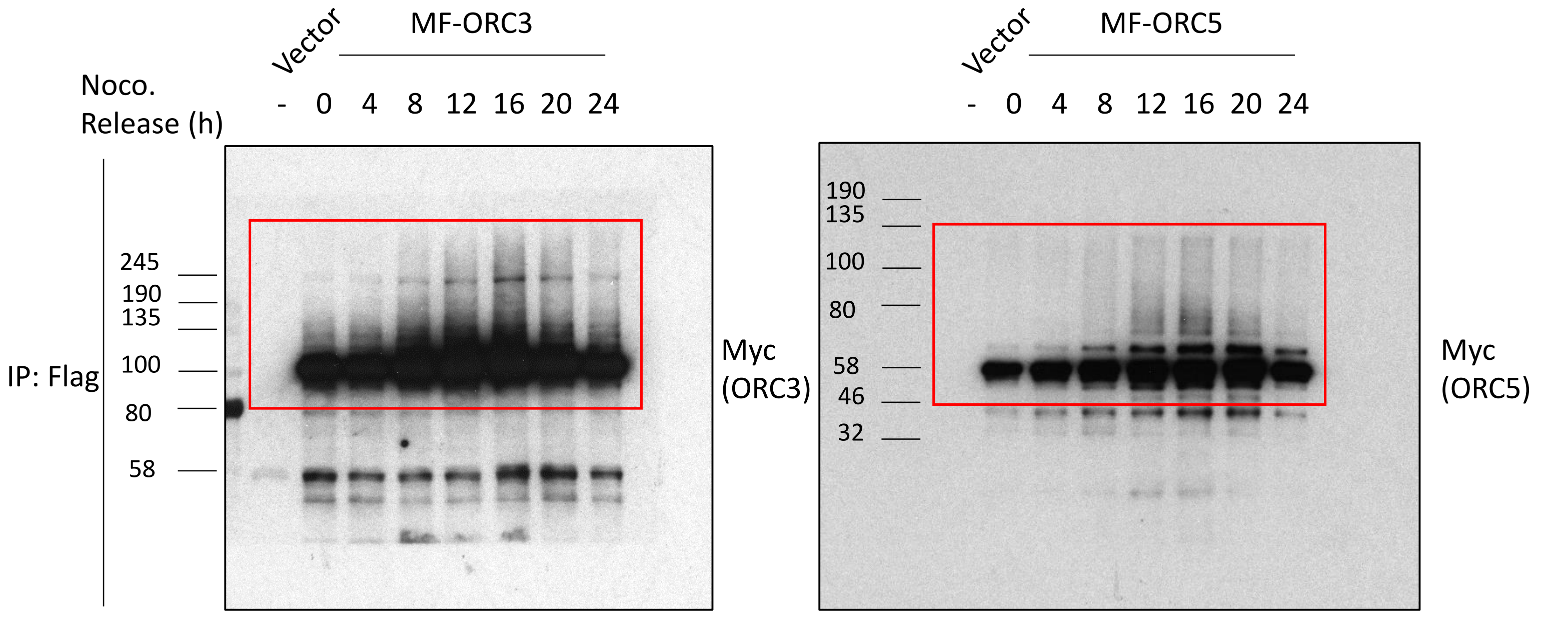

Supplementary Figure 12: Uncropped blots corresponding to Figure 3

SDS polyacrylamide gels for western blots found in Figure 3- a- b- c- d- e.
For Figure 3- a- b- c, gels were loaded with the same samples from the same experiment.
Boxed areas correspond to the images presented in the main text.

For Figure 4- a:

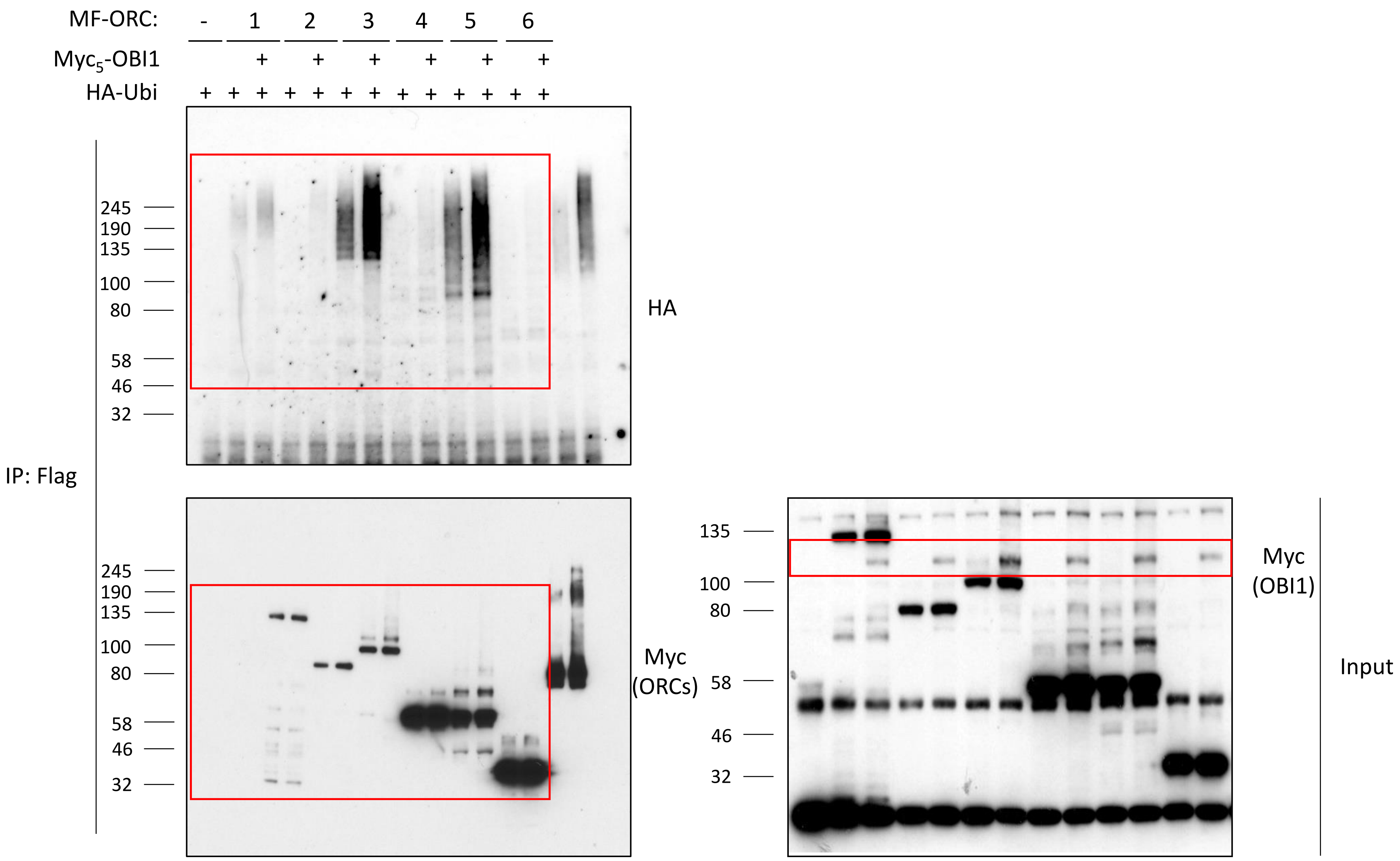

For Figure 4- b- c:

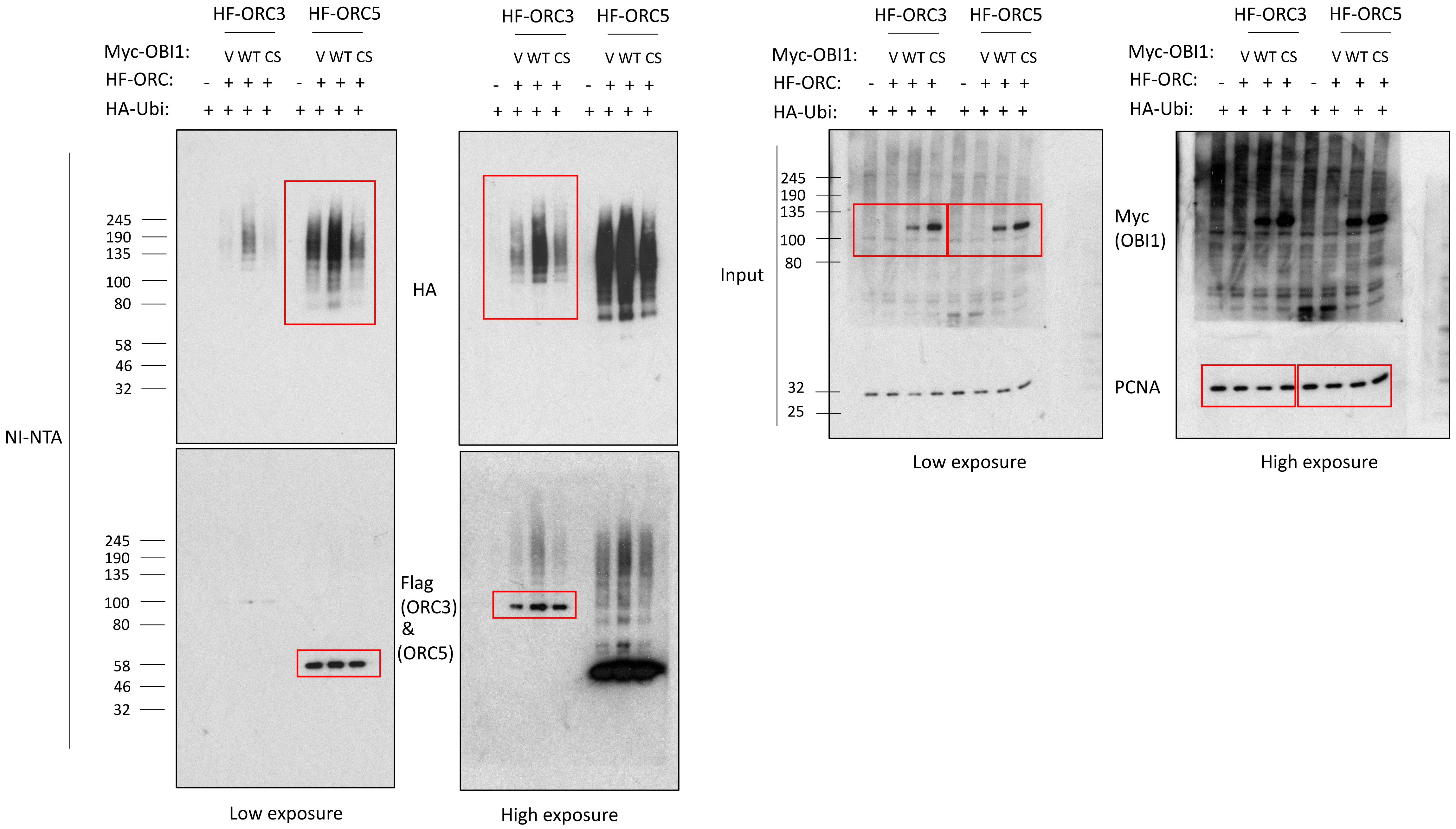

For Figure 4- d- e:

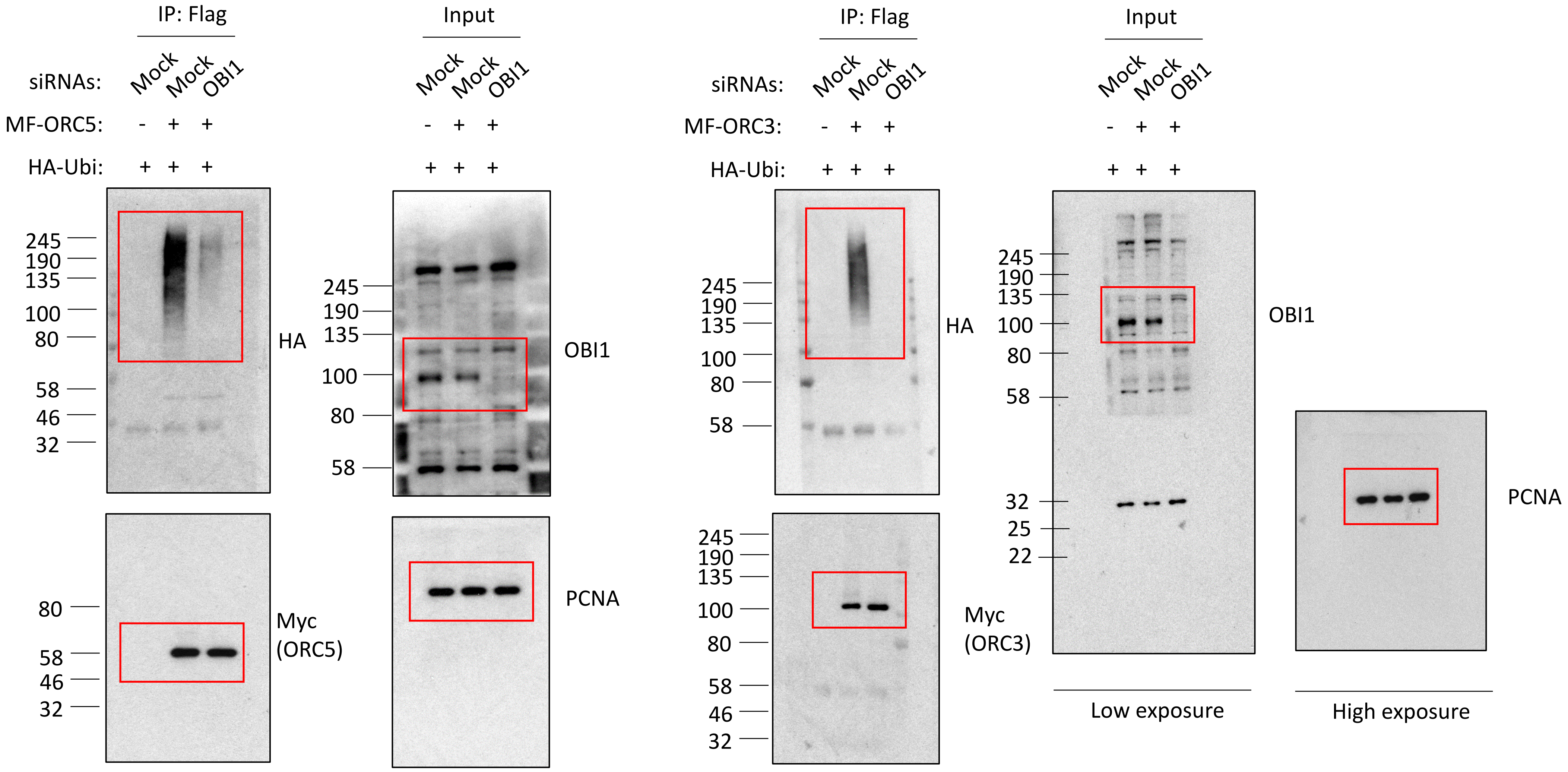

Supplementary Figure 13: Uncropped blots corresponding to Figure 4

SDS polyacrylamide gels for western blots found in Figure 4- a- b- c- d- e. Gels were loaded with the same samples from the same experiment.
Boxed areas correspond to the images presented in the main text.

For Figure 4- f:

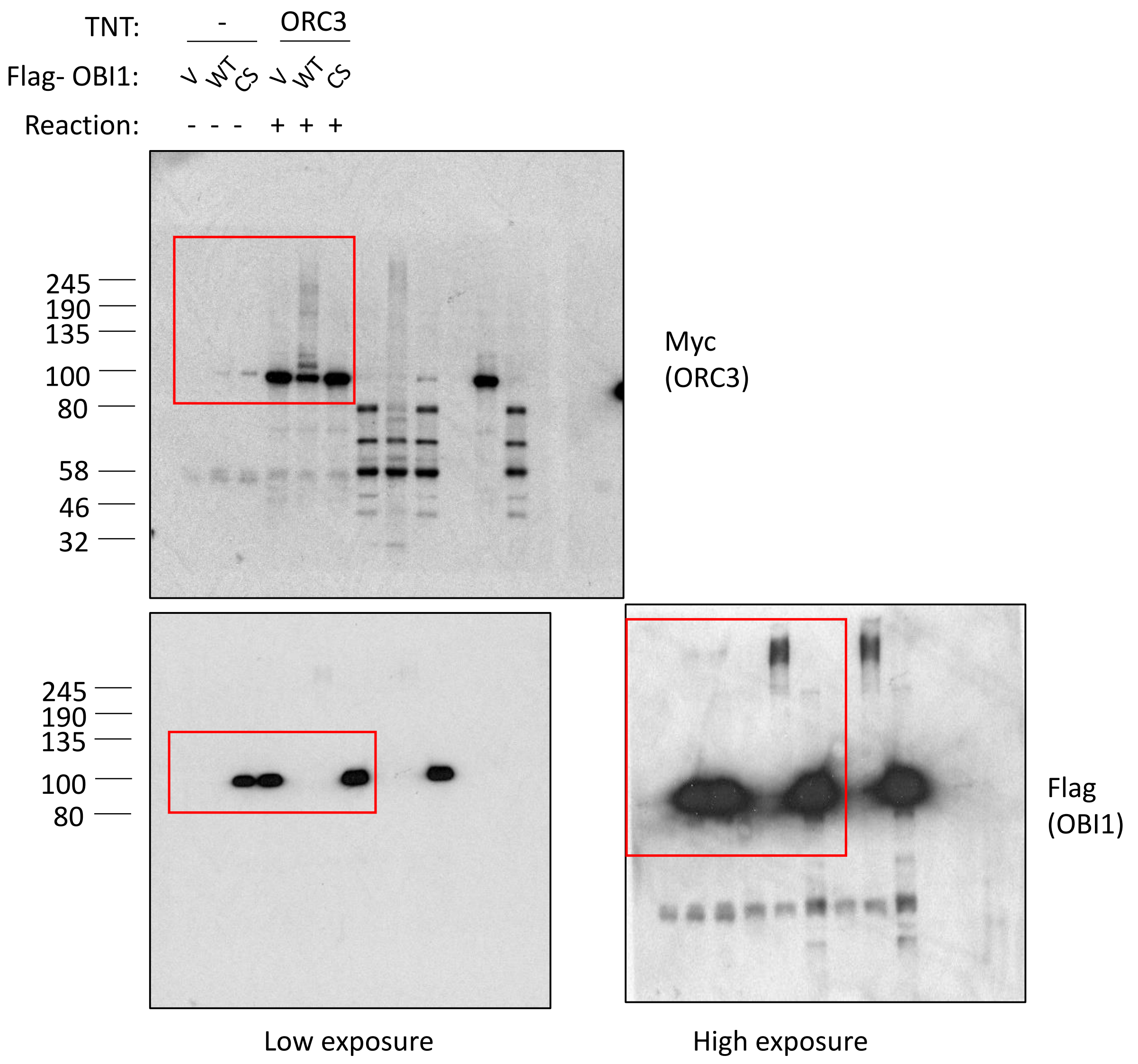

For Figure 4- g:

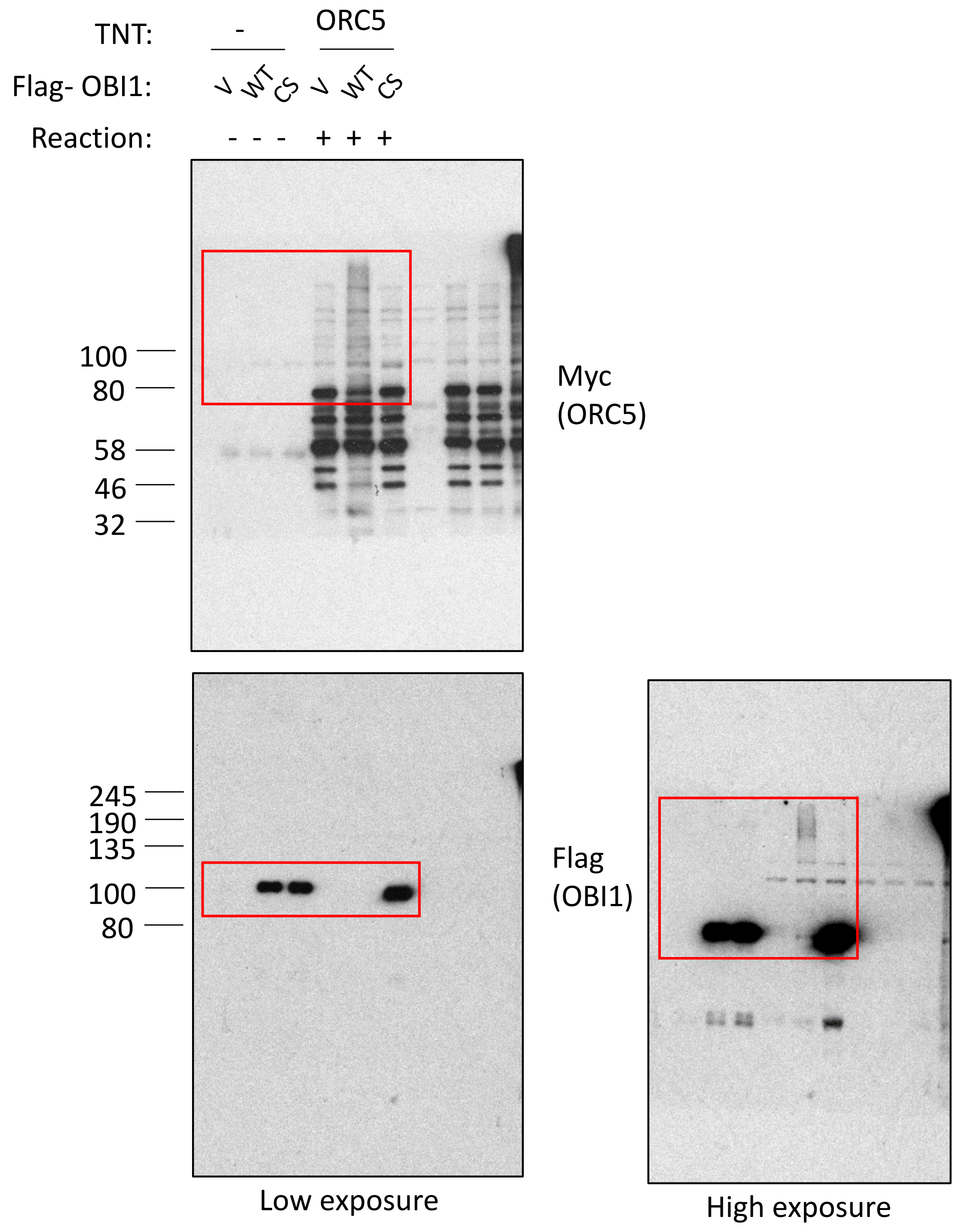

For Figure 4- h:

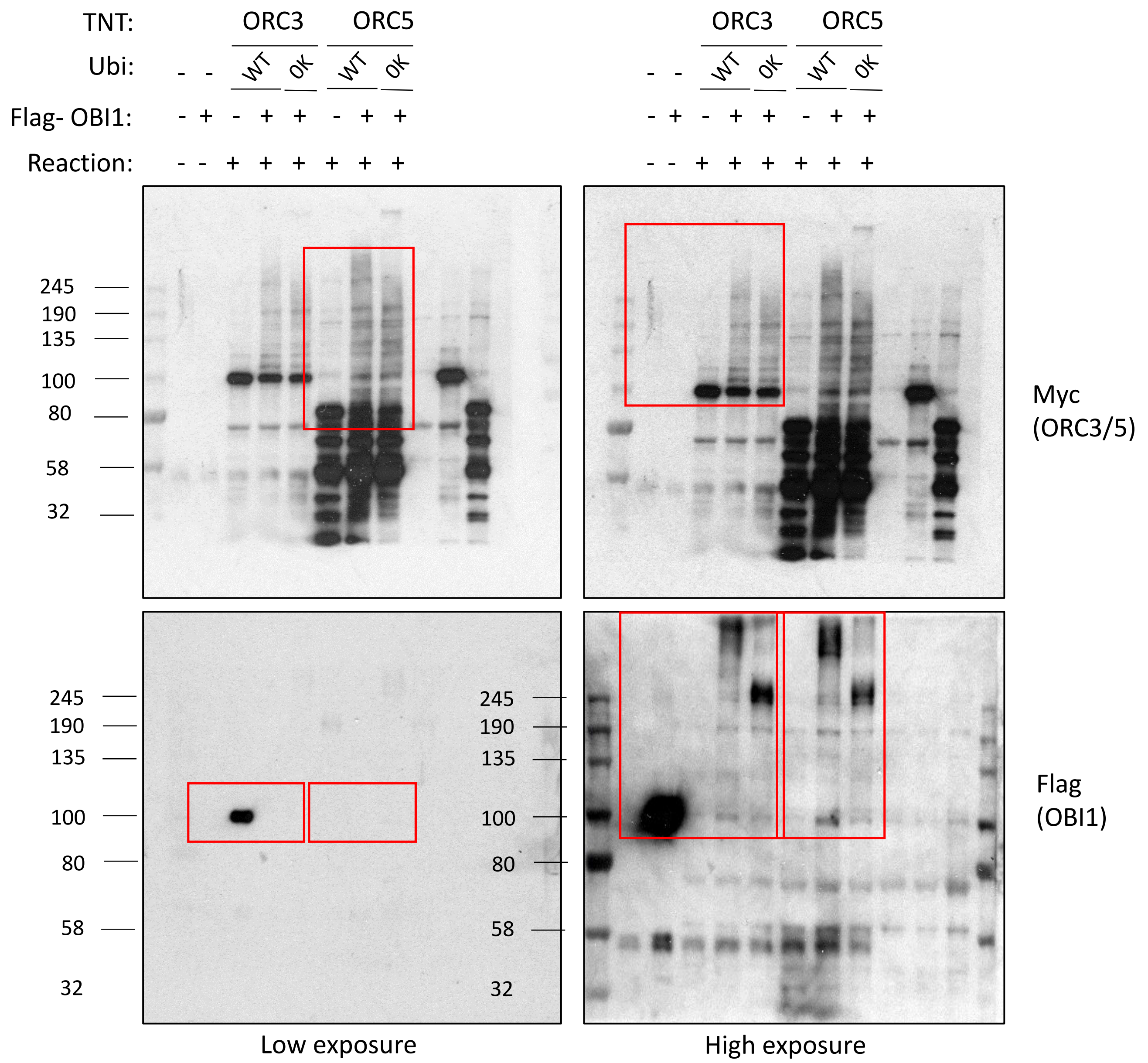

Supplementary Figure 14: Uncropped blots corresponding to Figure 4

SDS polyacrylamide gels for western blots found in Figure 4- f- g- h. Gels were loaded with the same samples from the same experiment.
Boxed areas correspond to the images presented in the main text.
