# Supplementary material for: The ORC ubiquitin ligase OBI1 promotes DNA replication origin firing
Source: Nat Commun. 2019 Jun 3;10:2426. doi: 10.1038/s41467-019-10321-x (PMC6547688; doi:10.1038/s41467-019-10321-x)
Supplement: Supplementary file 3 — Reporting Summary [file 41467_2019_10321_MOESM3_ESM.pdf]

nature research

Corresponding author(s): DBPR NCOMMS-18-17665B

Last updated by author(s): Apr 11, 2019

Reporting Summary

Nature Research wishes to improve the reproducibility of the work that we publish. This form provides structure for consistency and transparency in reporting. For further information on Nature Research policies, see Authors & Referees and the Editorial Policy Checklist.

Statistics

For all statistical analyses, confirm that the following items are present in the figure legend, table legend, main text, or Methods section.

n/a	Confirmed	
☐	☒	The exact sample size (*n*) for each experimental group/condition, given as a discrete number and unit of measurement
☐	☒	A statement on whether measurements were taken from distinct samples or whether the same sample was measured repeatedly
☐	☒	The statistical test(s) used AND whether they are one- or two-sided<br>*Only common tests should be described solely by name; describe more complex techniques in the Methods section.*
☒	☐	A description of all covariates tested
☒	☐	A description of any assumptions or corrections, such as tests of normality and adjustment for multiple comparisons
☒	☐	A full description of the statistical parameters including central tendency (e.g. means) or other basic estimates (e.g. regression coefficient) AND variation (e.g. standard deviation) or associated estimates of uncertainty (e.g. confidence intervals)
☒	☐	For null hypothesis testing, the test statistic (e.g. *F*, *t*, *r*) with confidence intervals, effect sizes, degrees of freedom and *P* value noted<br>*Give P values as exact values whenever suitable.*
☒	☐	For Bayesian analysis, information on the choice of priors and Markov chain Monte Carlo settings
☒	☐	For hierarchical and complex designs, identification of the appropriate level for tests and full reporting of outcomes
☒	☐	Estimates of effect sizes (e.g. Cohen's *d*, Pearson's *r*), indicating how they were calculated

Our web collection on statistics for biologists contains articles on many of the points above.

Software and code

Policy information about availability of computer code

Data collection	As listed in the Methods section:<br>For DNA combing: "Image acquisition using a fully motorized Leica DM6000 microscope equipped with a CoolSNAP HQ2 1 CCD camera and controlled by MetaMorph (Roper Scientific)".<br>For DNA spreading: "Images were acquired by immunofluorescence microscopy (Leica DM6000 or Zeiss ApoTome, RIO imaging facility) and a Coolsnap HQ CCD camera 25 (Photometrics)."
Data analysis	As listed in the Methods section:<br>For DNA combing: ". Inter-origin distances and replication fork speed were measured manually using the MetaMorph software. Statistical analyses of inter-origin distances and replication fork speed were performed using Prism 5.0 (GraphPad)."<br>For DNA spreading: " Images were analysed with the Metamorph software (Molecular Devices). Fork speed was measured in more than 200 forks using the ImageJ software"

For manuscripts utilizing custom algorithms or software that are central to the research but not yet described in published literature, software must be made available to editors/reviewers. We strongly encourage code deposition in a community repository (e.g. GitHub). See the Nature Research guidelines for submitting code & software for further information.

Data

Policy information about availability of data

All manuscripts must include a data availability statement. This statement should provide the following information, where applicable:

- Accession codes, unique identifiers, or web links for publicly available datasets
- A list of figures that have associated raw data
- A description of any restrictions on data availability

Data supporting the findings of this study are available within the article and its supplementary information files, including uncropped scans of the most important

blots. All data are available from the corresponding authors upon reasonable request.

Field-specific reporting

Please select the one below that is the best fit for your research. If you are not sure, read the appropriate sections before making your selection.

☒ Life sciences ☐ Behavioural & social sciences ☐ Ecological, evolutionary & environmental sciences

For a reference copy of the document with all sections, see nature.com/documents/nr-reporting-summary-flat.pdf

Life sciences study design

All studies must disclose on these points even when the disclosure is negative.

Sample size	The sample size for the experimental data was precised in the manuscript.
Data exclusions	No data was excluded.
Replication	All attempts at replication were successfull.
Randomization	The experiments were not randomized. For the analysis of fork density, images were acquired in an unbiased manner (randomly), without first searching for the presence of IdU-CldU tracts.<br>For soft-agar growth, colonies were photographed in randomly selected fields using a phase-contrast microscope.
Blinding	Investigators were not blinded to allocation during experiments and outcome assessment.

Reporting for specific materials, systems and methods

We require information from authors about some types of materials, experimental systems and methods used in many studies. Here, indicate whether each material, system or method listed is relevant to your study. If you are not sure if a list item applies to your research, read the appropriate section before selecting a response.

Materials & experimental systems

n/a	Involved in the study	
☐	☒	Antibodies
☐	☒	Eukaryotic cell lines
☒	☐	Palaeontology
☒	☐	Animals and other organisms
☒	☐	Human research participants
☒	☐	Clinical data

Methods

n/a	Involved in the study	
☒	☐	ChIP-seq
☐	☒	Flow cytometry
☒	☐	MRI-based neuroimaging

Antibodies

Antibodies used	The complete list of primary and secondary antibodies used, including supplier name, catalog number is provided in the Methods section "Reagents and Antibodies".
Validation	All the antibodies used were validated by the manufacturer and checked by us by Western blot.<br>Validation data are provided on the manufacturer's website, along with citation and references.

Eukaryotic cell lines

Policy information about cell lines

Cell line source(s)	Platinum-E cells were from Cell Biolabs<br>T98G cells, U2OS cells, and NIH 3T3 cells were kindly provided by Geneviève Rodier.<br>HeLa S3 cells were provided by Bijan Sobhian.
Authentication	The cell lines used were previously authenticated by the providers.
Mycoplasma contamination	All cell lines were regularly tested for mycoplasma contamination.
Commonly misidentified lines<br>(See ICLAC register)	N/A

Flow Cytometry

Plots

Confirm that:

☒ The axis labels state the marker and fluorochrome used (e.g. CD4-FITC).

☒ The axis scales are clearly visible. Include numbers along axes only for bottom left plot of group (a 'group' is an analysis of identical markers).

☒ All plots are contour plots with outliers or pseudocolor plots.

☒ A numerical value for number of cells or percentage (with statistics) is provided.

Methodology

Sample preparation	As listed in the Methods section: "For cell cycle analysis, ethanol-fixed cells were incubated with RNase A and propidium iodide, and analysed by flow cytometry using a MACSQuant cytometer (Miltenyi) as described 38. Alternatively, cells were pulsed with 100 nM BrdU for 15min prior to fixing. After HCl denaturation, BrdU incorporation was detected using a mouse anti-BrdU antibody followed by an anti-mouse FITC-coupled antibody. BrdU incorporation was quantified by measuring the fluorescence intensity of BrdU-positive cells. The value of control cells (siMock) was normalized to 100%. The fluorescence value of BrdU-positive cells from experimental conditions was expressed as the percentage of fluorescence relative to that of control cells. The summary graph is the mean of three independent experiments."
Instrument	MACSQuant cytometer (Miltenyi)
Software	MACSQuantify (version 2.6).
Cell population abundance	No cell sorting was performed.
Gating strategy	*Describe the gating strategy used for all relevant experiments, specifying the preliminary FSC/SSC gates of the starting cell population, indicating where boundaries between "positive" and "negative" staining cell populations are defined.*

☐ Tick this box to confirm that a figure exemplifying the gating strategy is provided in the Supplementary Information.
